# *Cis*-regulation and natural selection in the evolution of human DNA methylation

**DOI:** 10.7554/eLife.110575

**Published:** 2026-11-12

**Authors:** Zhenzhen Ma, Alexander L Starr, David Gokhman, Hunter B Fraser

**Affiliations:** 1 Department of Biology, Stanford University Stanford United States; 2 Department of Molecular Genetics, Weizmann Institute of Science Rehovot Israel; Max Planck Institute for Evolutionary Anthropology Germany; Max Planck Institute for Biology Tübingen Germany

**Keywords:** human, chimpanzee, methylation, *cis*-regulation, gene expression, evolution, Other

## Abstract

The vast collection of human-specific traits—such as our unique morphology, cognition, behavior, and diseases—has long been attributed to gene expression divergence between us and our closest living relatives, chimpanzees. Theory suggests that changes to *cis*-regulatory elements such as promoters and enhancers may drive evolutionary adaptation, and DNA methylation is a key factor in transcriptional *cis*-regulation. However, we still lack an understanding of (1) how species-specific methylation patterns arise; (2) their downstream effects; and (3) whether they are a common target of natural selection. In this study, we investigated these three questions. By combining a novel hypothesis testing framework with DNA methylation data from six human and chimpanzee cell types, as well as fused interspecies hybrid cells, we disentangled *cis*- vs. *trans*-acting methylation divergence across the genome. Across cell types, we found that methylation divergence is primarily driven in *cis*, which can be linked in some cases to nearby sequence variants such as CpG gains and losses. Although less common, regions with *trans*-acting methylation divergence were enriched for specific transcription factor (TF) binding motifs, suggesting a role of TFs such as FOXM1 in these differences. Having established these causes of methylation divergence, we then examined the functional consequences of differential methylation. Although methylation lacks a consistent relationship with transcription, we observed that associations between methylation and gene expression are stronger for genes with *cis*-regulatory divergence. Moreover, we identified lineage-specific selection shaping promoter methylation at the level of entire pathways, including those associated with human-specific traits such as speech, cognition, and susceptibility to infection with hepatitis C. Collectively, our findings provide a mechanistic framework suggesting that DNA methylation may occupy a key position, mediating the effects of both *cis*- and *trans*-acting factors on transcriptional networks, including those contributing to human-specific traits.

## Introduction

While gene expression divergence has long been considered the primary driver of human evolution, identifying the molecular mechanisms underlying uniquely human traits remains challenging, particularly when they involve epigenetic modifications that control cell-type-specific expression patterns (Fraser, 2013; King and Wilson, 1975; Reilly and Noonan, 2016; Romero et al., 2012). Theory suggests that cell-type-specific *cis*-regulatory variants may fuel evolutionary adaptation through their precise effects on individual genes within specific cell types, avoiding potentially deleterious consequences of broader *trans*-acting changes that affect multiple targets simultaneously (Prud’homme et al., 2007; Wittkopp and Kalay, 2012). However, while recent advances have enabled quantification of human-specific divergence in gene expression and chromatin accessibility, the role of DNA methylation—a key regulator of gene expression—in coordinating these changes and driving human-specific traits has not been fully explored (Wittkopp and Kalay, 2012; Blake et al., 2020; Kelley and Gilad, 2020; Ma et al., 2022; Mack and Nachman, 2017). DNA methylation, occurring primarily at CpG dinucleotides, is generally associated with transcriptional repression and provides stable, long-term regulatory control that has been linked to species-specific phenotypes in mammals (Jones, 2012). Comparative methylome studies between humans and chimpanzees have revealed widespread tissue-specific methylation divergence and linked human-specific hypomethylation to neurodevelopmental evolution and disease susceptibility (Blake et al., 2020; Pai et al., 2011; Gokhman et al., 2020; Zeng et al., 2012; Hernando-Herraez et al., 2015a; Hernando-Herraez et al., 2013; Hernando-Herraez et al., 2015b; Mendizabal et al., 2016; Fukuda et al., 2013; Enard et al., 2004; Banerjee et al., 2019; Guevara et al., 2020).

**Figure 1.**
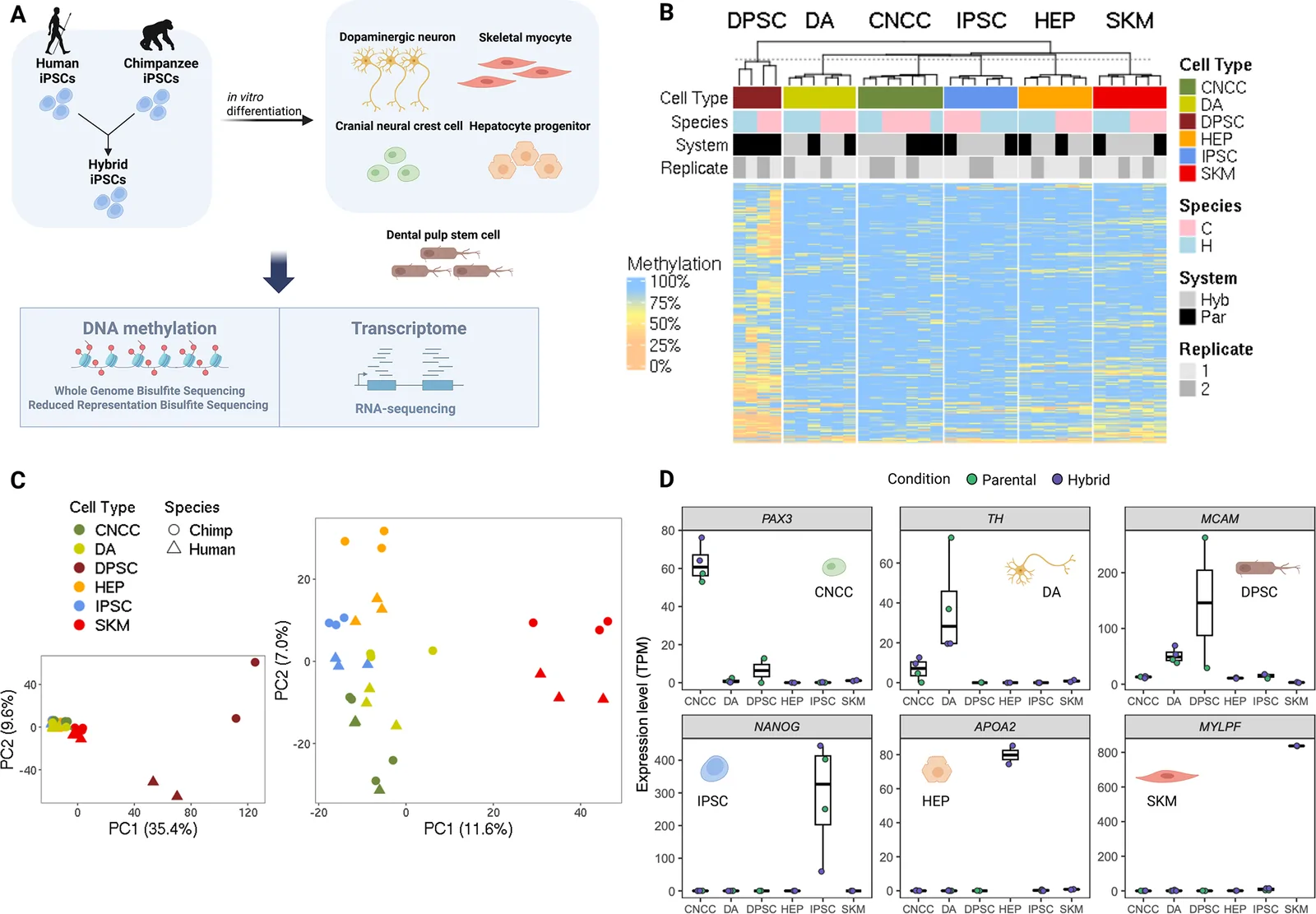
DNA methylation profiles across diverse human, chimpanzee, and hybrid cell types. (**A**) Four cell types were differentiated from human, chimpanzee, and hybrid induced pluripotent stem cells (iPSCs). The cell types span diverse body systems, including skeletal myocytes for the musculoskeletal system, dopaminergic neurons for the central nervous system, cranial neural crest cells for craniofacial development, and hepatocyte progenitors for the liver. We collected DNA methylation and RNA-sequencing data from these cell types from humans, chimpanzees, and their hybrids. Dental pulp stem cells (DPSCs) were also collected from human and chimpanzee adult tissues. (**B**) Clustering analysis and (**C**) principal component analysis (PCA) between samples for all cell types (left) and for all iPSC-derived cell types (i.e. excluding DPSC, right). Parent/hybrid stratifications are shown separately in Figure 1—figure supplement 1. Each replicate represents an independent biological differentiation from the indicated iPSC line. (**D**) Cell-type-specific markers of gene expression. Created with BioRender.com.

Studies of allele-specific expression (ASE) and chromatin accessibility measurements in interspecies hybrids provide a powerful approach for studying regulatory evolution by eliminating confounding factors inherent to cross-species comparisons, including differences in cell-type composition, environmental conditions, and developmental timing (Agoglia et al., 2021; Gokhman et al., 2021; Song et al., 2021; Wang et al., 2024b; Starr et al., 2023; Song et al., 2025; Barr et al., 2023; Starr and Fraser, 2025; Mack et al., 2023; Hu et al., 2022). Analogously, allele-specific methylation (ASM) in human-chimpanzee hybrid cells enables the isolation of *cis*-regulatory effects while controlling for shared *trans*-acting factors. *Cis*-regulation of methylation is caused by factors such as sequence variants affecting methylation on the same allele as the variants, typically acting locally. In contrast, *trans*-regulation of methylation involves diffusible factors such as DNA methyltransferases, transcription factors (TFs), or chromatin modifiers that can influence methylation patterns across many genomic loci (Figure 1A). Studying ASM enables direct quantification of the relative contribution of local vs. diffusible factors to species-specific methylation patterns.

**Figure 1—figure supplement 1.**
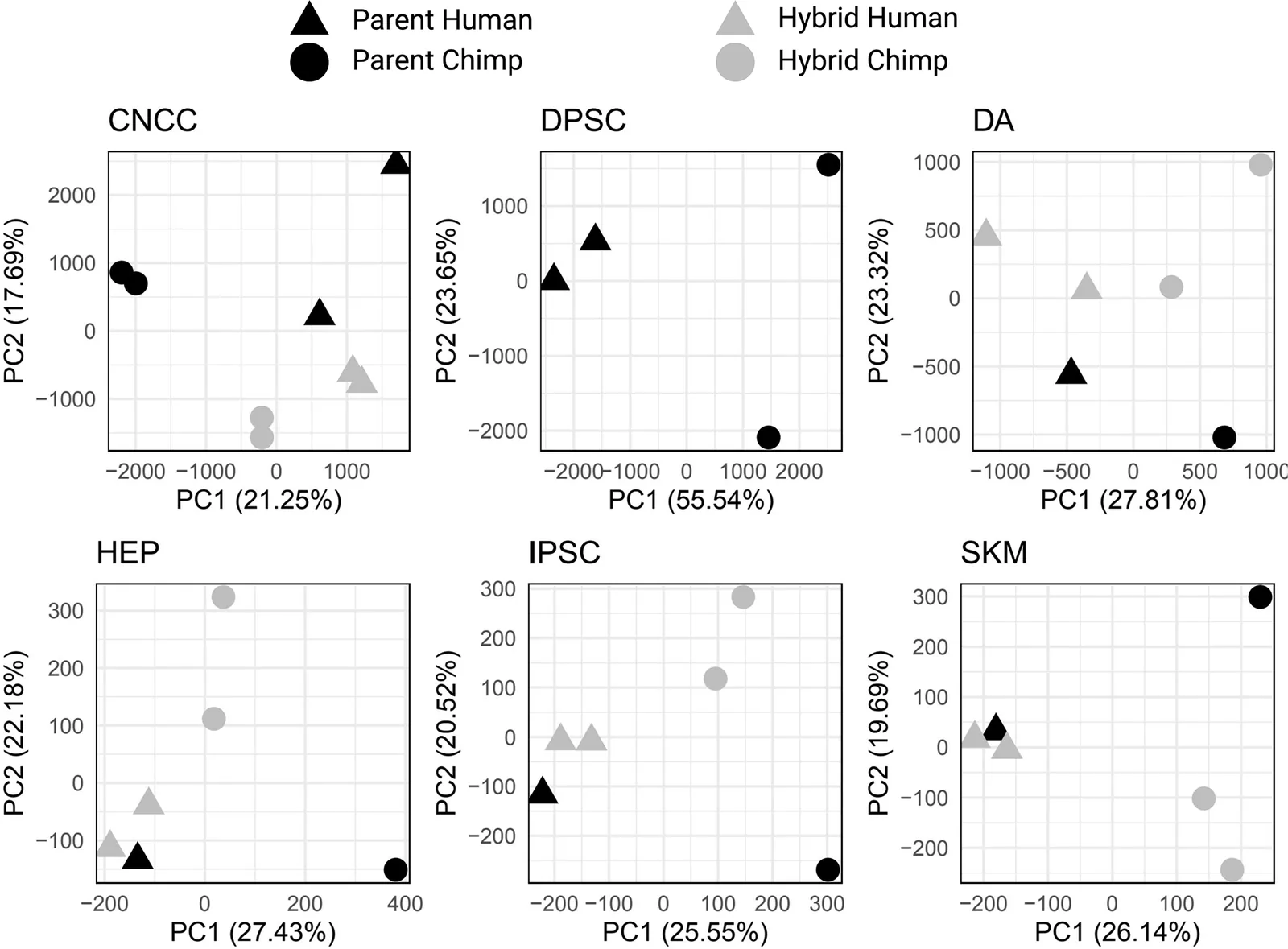
Principal component analysis (PCA) on CpG methylation from BS-seq for individual cell types. Species are clearly separated by PC1, and systems (parent and hybrid) are separated by PC2 in all cell types.

**Figure 1—figure supplement 2.**
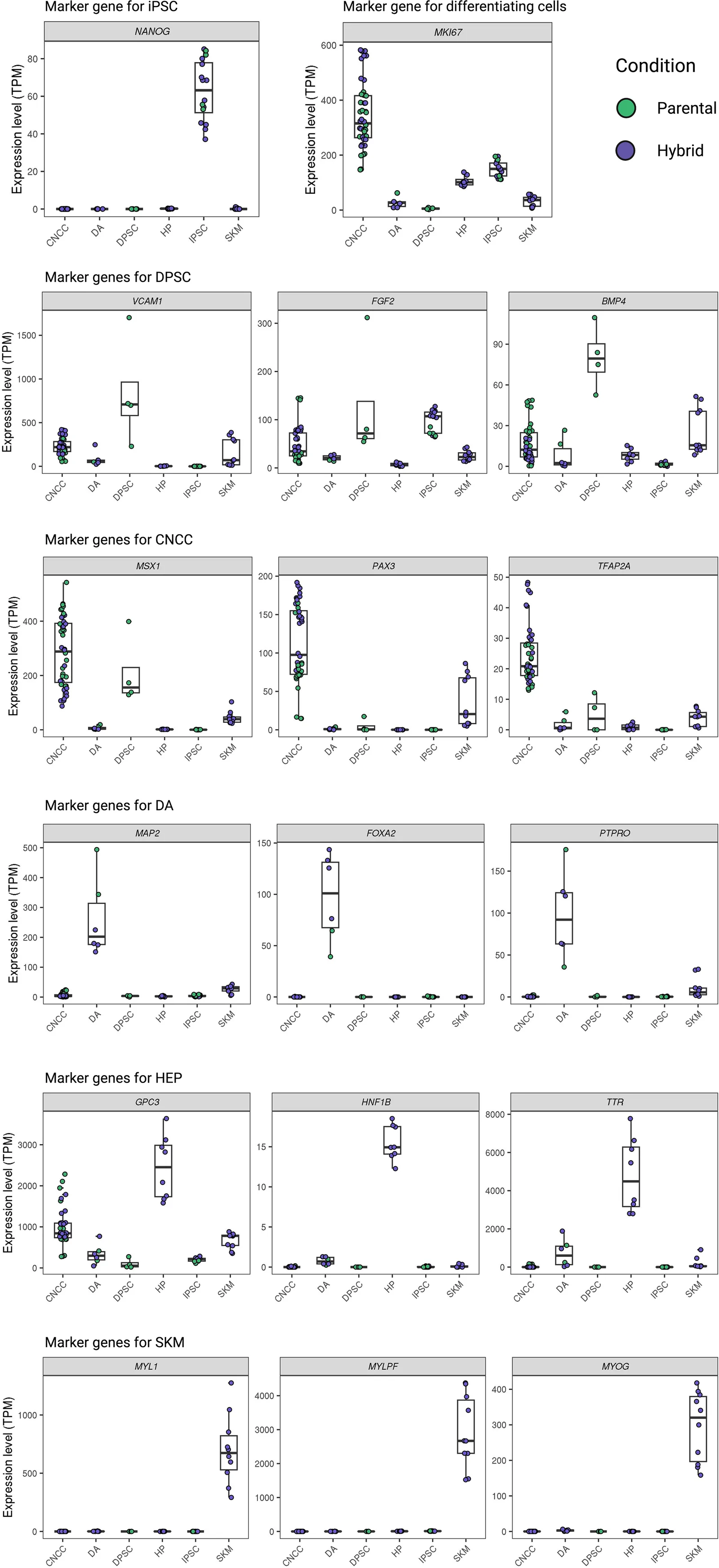
Expression of cell-type-specific marker genes in different cell types. TPM: transcript per million.

While previous studies of primate methylation evolution in postmortem tissues and blood have provided valuable foundational insights (Blake et al., 2020; Pai et al., 2011; Gokhman et al., 2020; Zeng et al., 2012; Hernando-Herraez et al., 2015a; Hernando-Herraez et al., 2013; Mendizabal et al., 2016; Fukuda et al., 2013; Enard et al., 2004; Banerjee et al., 2019; Guevara et al., 2020; Vilgalys et al., 2019; Housman et al., 2021), several important questions remain to be addressed. These include: (1) generating comprehensive genome-wide methylation maps across multiple species and cell types; (2) distinguishing between *cis*- vs. *trans*-regulatory mechanisms underlying species-specific methylation differences; (3) identifying *cis*-acting genetic variants and *trans*-acting factors underlying these differences; (4) identifying lineage-specific natural selection on methylation; and (5) linking this selection to gene expression and candidate traits. Addressing these gaps would enhance our understanding of how genetic variants impact methylation and in turn shape adaptive traits.

Human-chimpanzee tetraploid hybrid induced pluripotent stem cells (iPSCs), generated through in vitro cell fusion, provide a controlled experimental system for quantifying *cis*- and *trans*-regulatory contributions to methylation divergence by hosting both species’ alleles in an identical *trans*-environment. By measuring ASM and ASE across multiple cell types, this system generates genome-wide maps of cell-type-specific regulatory differences that may contribute to evolutionary adaptation. In this study, we generated bisulfite sequencing data from human, chimpanzee, and hybrid iPSCs, cranial neural crest cells (CNCCs), dopaminergic neurons (DAs), skeletal myocytes (SKMs), and hepatocyte progenitors (HEPs), as well as from primary human and chimpanzee dental pulp stem cells (DPSCs). We combined these data with previously published (Wang et al., 2024b) and newly generated RNA-sequencing data from all six cell types.

Using these data, we investigated the causes of methylation divergence and quantified contributions of *cis*- and *trans*-acting mechanisms that establish interspecies differences in methylation patterns. We developed a hypothesis testing framework tailored to count-based proportional methylation data and extended a changepoint detection algorithm to identify differentially methylated regions (DMRs) under consistent *cis*- or *trans*-regulation. We show that *cis*-acting mechanisms predominantly drive genome-wide methylation divergence, with *cis*-regulated DMRs likely affected by nearby species-specific single-nucleotide variants. In contrast, *trans*-regulated DMRs were enriched for TF binding motifs, including pioneer factors and chromatin remodelers that may mediate *trans*-acting divergence. Concordance between ASM and ASE across cell types revealed species-specific and context-dependent regulatory relationships linking methylation to gene expression. Finally, coordinated methylation-expression divergence in gene sets related to speech, cognition, and viral infection susceptibility connected lineage-specific selection to candidate phenotypes. In sum, our work provides a framework for understanding how divergent *cis*- and *trans*-acting factors converge on methylation patterns to produce species-specific traits.

## Results

### Measurement of DNA methylation across diverse cell types

To establish comprehensive methylation landscapes across evolutionarily relevant cell types, we differentiated iPSCs from two humans, two chimpanzees, and two fused tetraploid hybrids into four cell types (see Materials and methods) (Gokhman et al., 2021; Wang et al., 2024b). We included an additional cell type, DPSCs, which are primary cells isolated from two human and two chimpanzee donors (Gokhman et al., 2021). We then generated whole-genome bisulfite sequencing (WGBS) data from human and chimpanzee parental and hybrid CNCCs and DAs, as well as parental DPSCs, complemented by reduced representation bisulfite sequencing (RRBS) data from iPSCs, SKMs, and HEPs (Figure 1A). For cross-cell-type analyses combining WGBS and RRBS data, we restricted to CpG sites covered in all samples after phasing, totaling 561,762 shared CpG sites. For hybrid samples, reads were assigned to the human or chimpanzee allele using species-specific single nucleotide variant (SNV) positions; on average, 51% ± 0.4% of all aligned reads were allele-resolved across hybrid WGBS samples (DA and CNCC), and 10.6% ± 0.2% of aligned reads were allele-resolved across hybrid RRBS samples (iPSCs, SKMs, and HEPs). The lower rate in RRBS reflects its short read lengths and its enrichment for CpG-dense promoter regions, which are evolutionarily conserved and therefore depleted of informative human-chimpanzee SNVs (see ‘RNA-sequencing and ASE analysis’ in Materials and methods section). Principal component analysis (PCA) and hierarchical clustering of methylation patterns showed that samples clustered primarily by cell type, then by species, and finally by parental (human and chimpanzee) vs. hybrid system (Figure 1B, C, Figure 1—figure supplement 1). CNCC samples displayed a unique clustering pattern where parental vs. hybrid samples clustered separately, though examination of the dendrogram indicated this was due to a single human parental sample being a slight outlier (Figure 1B). The epigenetic distinctiveness of DPSCs relative to iPSC-derived cell types likely reflects their status as primary cells, which retain the epigenetic and transcriptional signatures of their tissue of origin and have not undergone reprogramming (Figure 1C). Known cell-type-specific gene expression markers validated the correct identity of each differentiated cell type (Figure 1D, Figure 1—figure supplement 2; Silva et al., 2009; Gerdes et al., 1984; Hatori et al., 2023; Svandova et al., 2023; Bai et al., 2023; Soldatov et al., 2019; Bronner and Simões-Costa, 2016; Cordero et al., 2011; Metzakopian et al., 2012; Cassimeris and Spittle, 2001; Xu et al., 2022; Bockenhauer and Jaureguiberry, 2016; Grozdanov et al., 2006; Graffmann et al., 2022; Rubenstein et al., 2020). Overall, these results are consistent with our expectation that cell type is the predominant determinant of DNA methylation patterns, while species explains a much smaller proportion (Blake et al., 2020). In other words, a human HEP is much more similar to a chimpanzee HEP than it is to a human DA.

### Contribution of *cis-* and *trans*-regulation to DNA methylation divergence between human and chimpanzee

**Figure 2.**
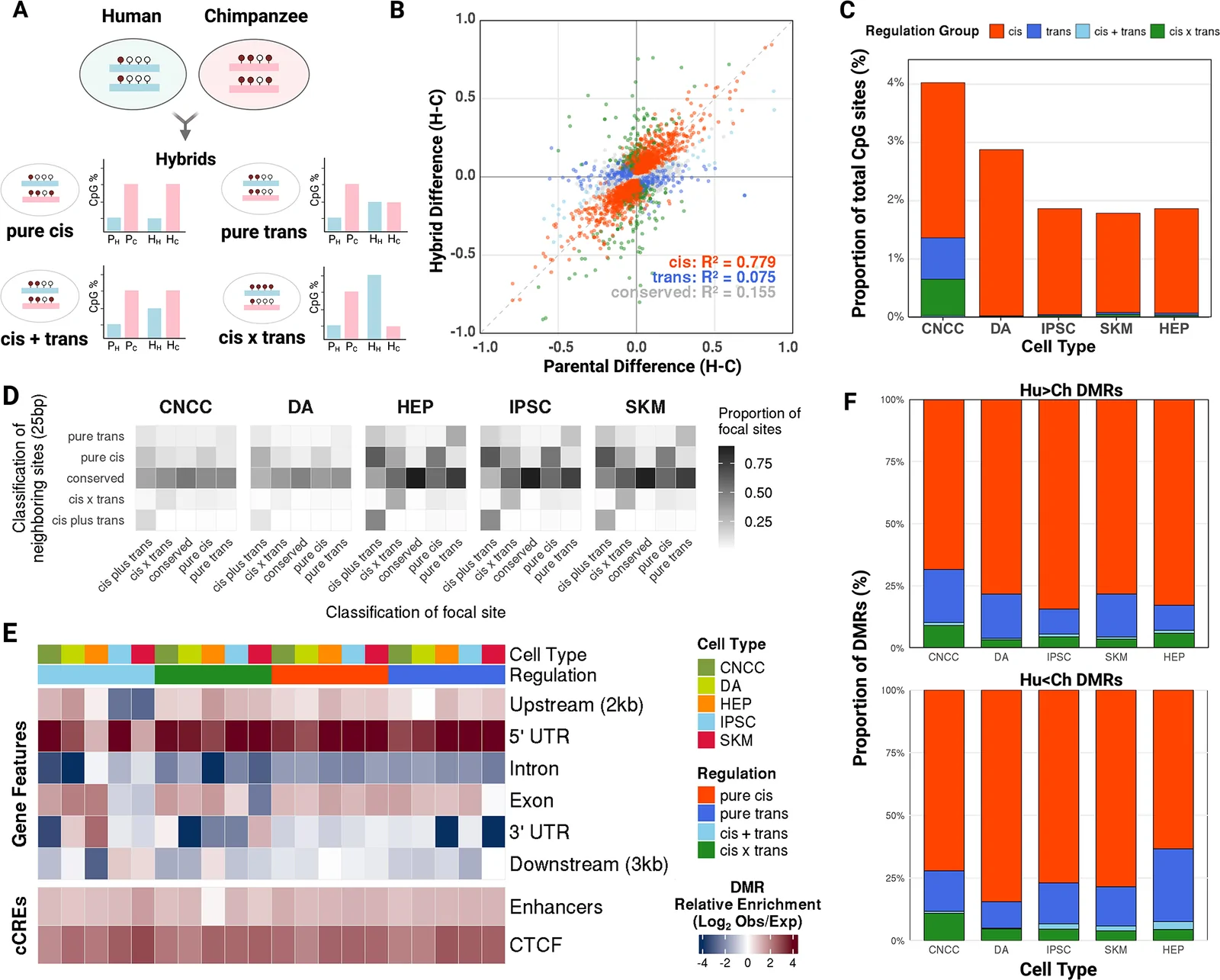
Contribution of *cis*- and *trans*-regulation to DNA methylation divergence between human and chimpanzee. (**A**) Interspecies differences in methylation levels per CpG site are compared between parental and hybrid systems and assigned a regulation category based on hypothesis testing. (**B**) Parental and hybrid methylation differences in cranial neural crest cell promoters and the respective regulation category at false discovery rate (FDR)<0.05 (color key in panel (**C**)). Parental and hybrid methylation differences are plotted as the per-sample average difference of fractional methylation. (**C**) Quantification of relative contribution of non-conserved regulation groups to whole-genome CpG sites across cell types at FDR<0.05. (**D**) Heterogeneity of the distribution of CpG sites of all regulation groups. (**E**) Quantification of CpG regulatory clusters by overlap with genomic features of transcripts genome-wide as well as *cis*-regulatory elements, including promoters, enhancers, and CTCF-bound *cis*-regulatory regions (CTCF). (**F**) Quantification of relative contribution of non-conserved regulation groups to regulatory clusters across cell types (color key in panel (**C**)). Created with BioRender.com.

To understand the molecular mechanisms underlying species-specific methylation patterns, we first established a framework for distinguishing between *cis*- and *trans*-regulatory control of DNA methylation. By comparing methylation levels between human and chimpanzee alleles within the same cellular environment (hybrid cells), we can isolate *cis* effects from *trans* effects and determine which mechanism predominates in establishing species-specific methylation patterns (Figure 2).

**Figure 2—figure supplement 1.**
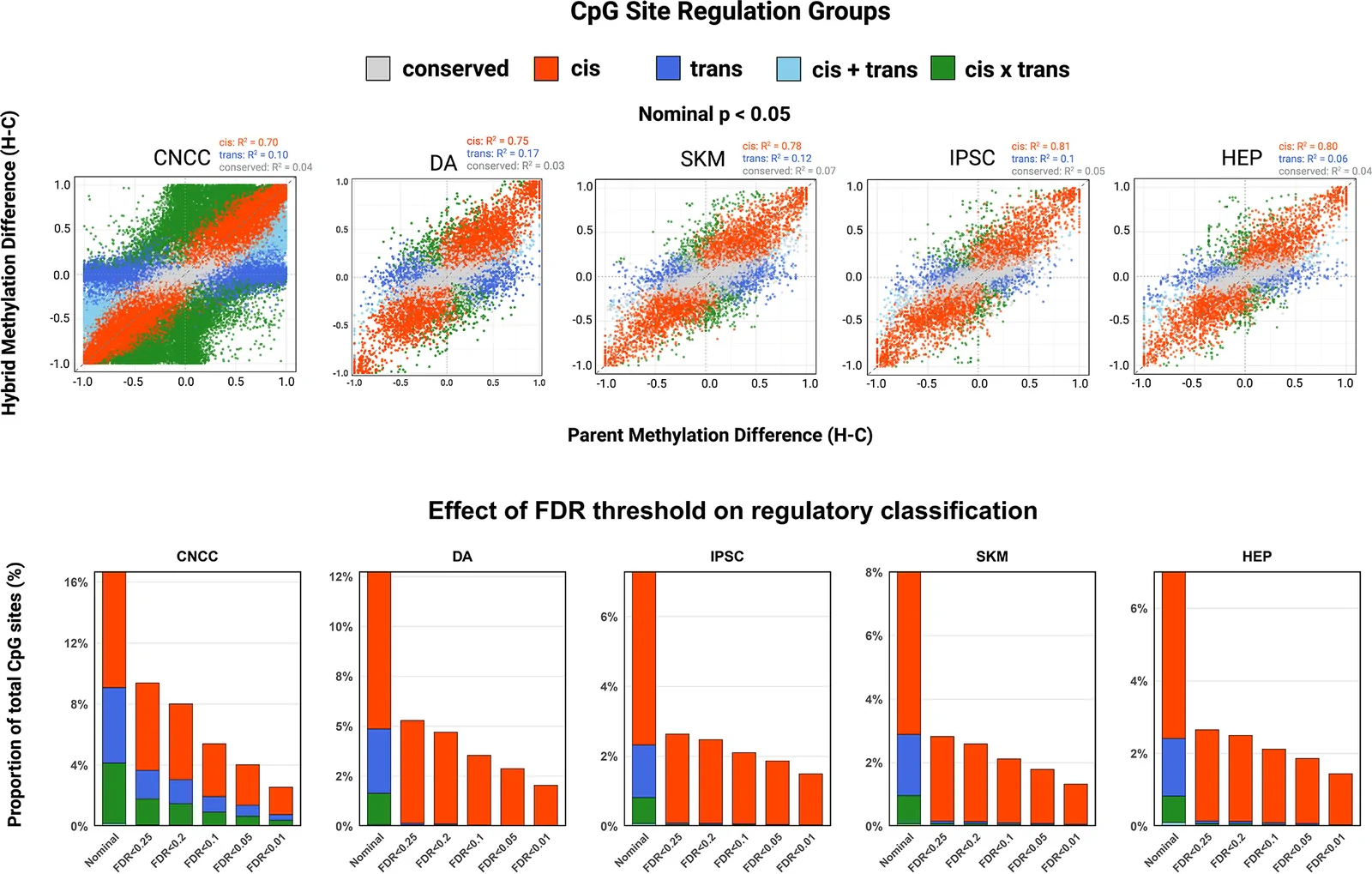
Characterization of regulation groups of all CpG sites for individual cell types. The methylated read counts and unmethylated read counts of each individual CpG site in hybrid and parents are used in the beta-binomial model to classify into regulation groups. Methylation difference is calculated as the difference of fractional methylation between orthologous CpG sites in human and chimpanzee. Cranial neural crest cell (CNCC) has more CpG sites than other cell types due to its high coverage. Scatterplot visualizes CpG site classification at nominal p<0.05 before false discovery rate (FDR) correction. After FDR correction, the majority of significant hits are *cis*-regulated CpG sites. Bar plot visualizes the percentages of CpG sites that fall into each regulation group at varying FDR cutoffs; conserved promoters are used in the calculation but not shown for clarity. For detailed statistics, see Supplementary file 1.

**Figure 2—figure supplement 2.**
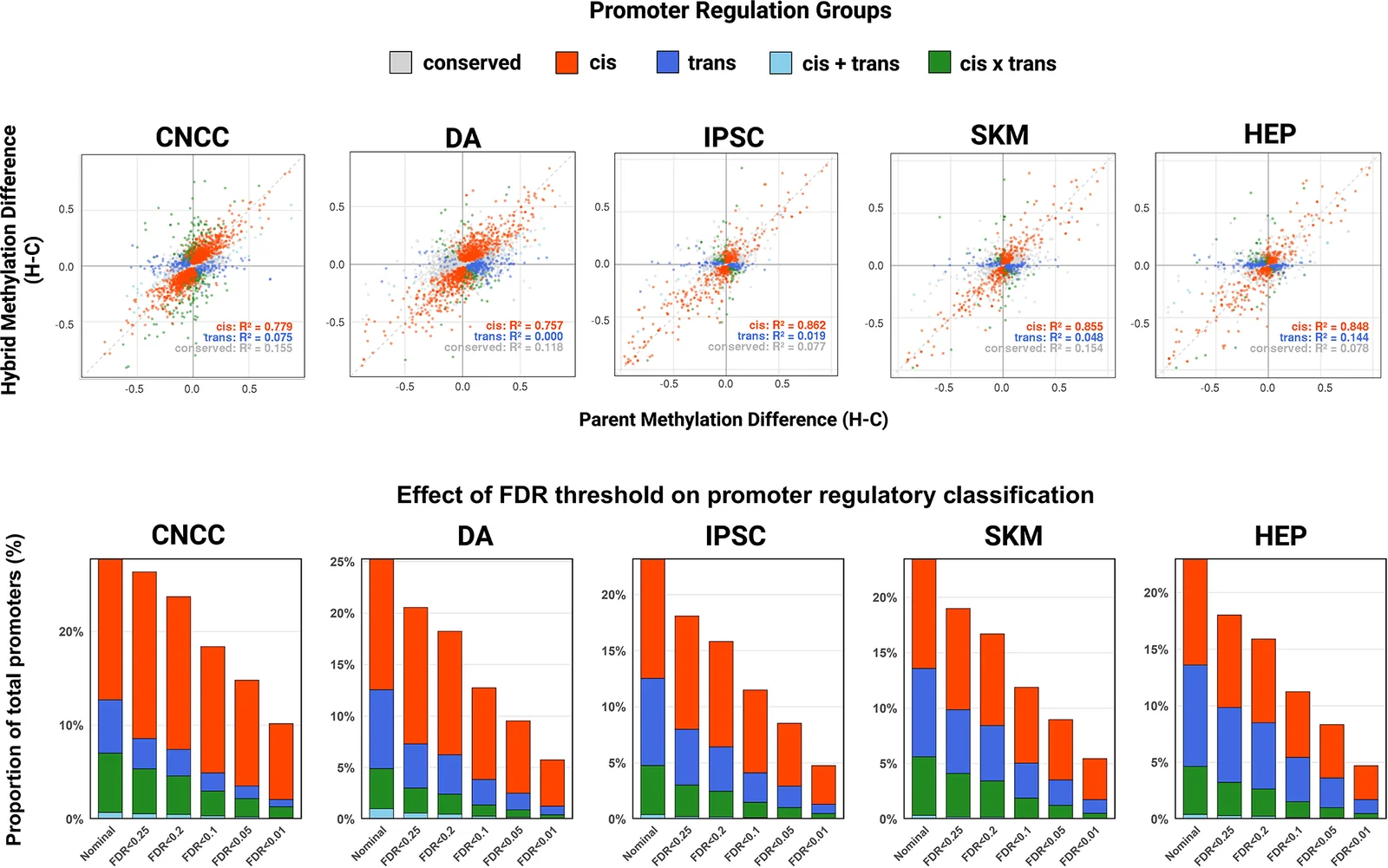
Characterization of regulation groups of all promoters for individual cell types. The pooled methylated and unmethylated read counts in hybrid and parents of each ENCODE promoter region are used in the beta-binomial model to classify into regulation groups. Promoter methylation was quantified as pooled fractional methylation, calculated as the total number of methylated cytosines divided by the total coverage across all CpG sites within each promoter. Scatterplot visualizes promoter classification at false discovery rate (FDR)<0.05. Bar plot visualizes the percentages of promoters that fall into each regulation group at varying FDR cutoffs; conserved promoters are used in the calculation but not shown for clarity. See Supplementary file 2 for detailed statistics.

**Figure 2—figure supplement 3.**
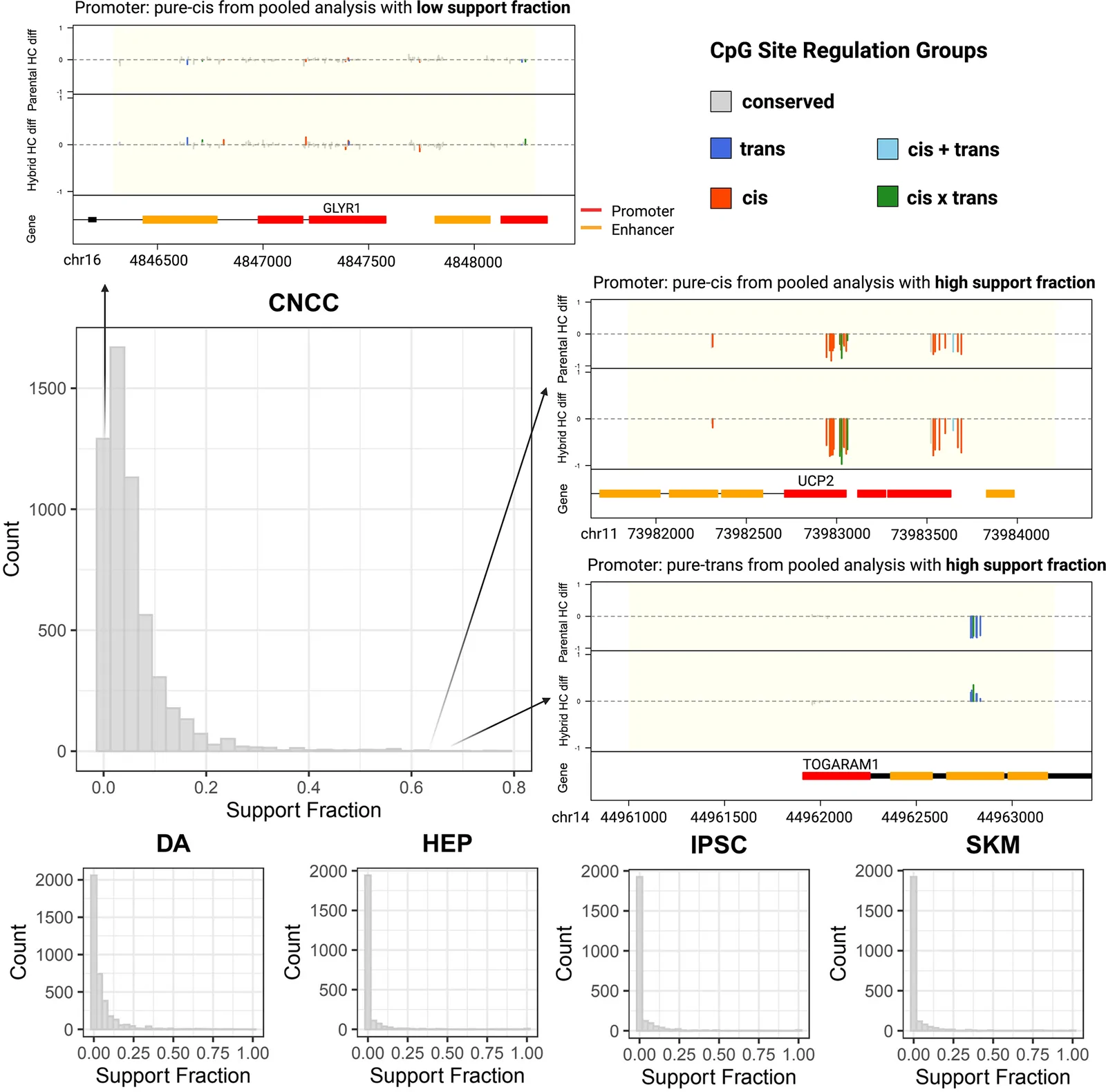
Pooling CpG sites across large genomic regions introduces noise and masks true regulatory patterns. The distribution of support fractions of non-conserved ENCODE-annotated promoters for all cell types showed that most promoter regions have heterogeneous classifications at single CpG site resolution (support fraction: fraction of individual CpG sites out of all sites with the same regulation classification as pooled promoter methylation). Within homogeneous promoters (promoters with high support fractions like *UCP2* and *TOGARAM1*), the majority of CpG sites have the same classification as the promoter. Within heterogeneous promoters (promoters with low support fractions like *GLYR1*), the majority of CpG sites have alternative classifications. Increasing region size results in the aggregation of multiple regulatory units with regions lacking coherent regulatory clusters, which leads to underestimation of differences between contributions of regulatory classifications and masks the true underlying regulatory signal. This highlights the importance of identifying differentially methylated regions (DMRs) exhibiting minimal classification heterogeneity using changepoint detection to prioritize signatures of regulation. See Supplementary file 2 for detailed statistics.

To quantify the relative contributions of *cis*- vs. *trans*-acting factors in establishing human-chimpanzee methylation differences, we applied a beta-binomial model framework adapted from Hallgrímsdóttir et al., 2024, to classify DMRs based on allele-specific patterns in hybrid vs. parental samples. To determine whether a CpG site reflects a difference in methylation due to *cis*- or *trans*-regulation, two hypothesis tests are used: one to evaluate the null hypothesis that the difference in methylation is due solely to *trans*, and the other solely to *cis* (see Materials and methods). Each site is assigned to one of five mutually exclusive regulatory categories. *Cis*-only sites show allelic differences in hybrids that are also preserved in the parental comparison. *Trans*-only sites show allelic differences in parentals that are absent in hybrids. *Cis+trans* (additive) sites show allelic differences in both systems, with the same species methylated higher in both, but with the magnitude of interspecies difference reduced in hybrids, indicating a partial *trans* contribution acting in the same direction as *cis. Cis*×*trans* sites show significant *cis* and *trans* effects acting in opposite directions. In these sites the interspecies difference is either larger in hybrids than in parentals, or reversed in sign, with one species more methylated in parentals and the other in hybrids. Conserved sites show no significant allelic differences in either system (Figure 2A and B). We investigated genome-wide methylation divergence by fitting the model to methylation levels at each CpG site (totaling 49,049,052 CpG sites across all cell types; see Supplementary file 1 for detailed statistics). In all cell types examined, most CpG sites (83–93%) had conserved patterns of methylation. Among divergent sites, nominally classified *cis*-regulation was over twice as common as *trans*-regulation (Figure 2—figure supplement 1, Supplementary file 1). At false discovery rate (FDR)<0.05, *cis*-regulation accounted for a mean of 91% of divergent sites and *trans*-regulation a mean of 4.5% across cell types, and *cis* remained the dominant category at every FDR cutoff tested (FDR<0.25–0.01; Figure 2C, Figure 2—figure supplements 1 and 2, Supplementary files 1 and 2). The genome-wide predominance of *cis*-regulation was further supported at the regional level (Figure 2B, Figure 2—figure supplements 1 and 3). This genome-wide predominance of *cis*-regulation indicates that local sequence differences between human and chimpanzee genomes are the primary drivers of methylation divergence, consistent with previous hypotheses of gene regulation that *cis*-regulatory changes may be less disruptive than broad *trans*-acting modifications (Fraser, 2013; King and Wilson, 1975; Reilly and Noonan, 2016; Romero et al., 2012; Wittkopp and Kalay, 2012).

The regional distribution of sites from different regulation groups reveals a consistent pattern across cell types: conserved CpG sites are most likely to be near other conserved sites, while divergent CpG sites are likely to be near conserved sites as well as sites within the same regulation group (Figure 2D). This suggests that CpG sites within the same regulation group tend to form coherent clusters in the genome, and divergent CpG clusters typically have conserved sites scattered within. When conducting regional analyses, methylation data is usually pooled from multiple sites within a pre-defined genomic region (Wittkopp and Kalay, 2012; Blake et al., 2020; Kelley and Gilad, 2020; Ma et al., 2022; Mack and Nachman, 2017). However, this regional heterogeneity can affect classification as well as estimates of how much each regulatory mechanism contributes to divergence between species (Figure 2—figure supplements 2 and 3, Supplementary file 2, see Materials and methods). Therefore, pooling data across regions should be approached carefully, as subsets of CpG sites within the same regulatory element can be controlled by distinct mechanisms. These findings underscore the importance of studying methylation divergence at high resolution.

To identify DMRs exhibiting consistent inter-species methylation directionality and minimal classification heterogeneity of regulation groups, we implemented a changepoint detection method with a tolerance threshold for regulatory homogeneity (see Materials and methods). This method identifies genomic loci where co-localized CpG sites demonstrate uniform regulatory behavior punctuated by a pre-defined fraction of heterogeneous sites: non-conserved classifications showing consistent directional methylation bias and conserved sites maintaining equal methylation levels between species. This method prioritizes DMRs that are controlled by the same mechanism and display coordinated methylation patterns (Supplementary file 3), thereby enriching for loci with interpretable regulatory mechanisms. Notably, DMRs identified through this method are enriched in 5' UTRs (just downstream of each transcription start site [TSS]) and CTCF-bound regions, supporting their functional relevance in gene regulation (Figure 2E). In total, we identified 9159 *cis*-DMRs and 2046 *trans*-DMRs across all cell types (see Materials and methods and Supplementary file 3). The quantification of *cis*- vs. *trans*-DMRs yielded the same conclusion that *cis*-regulation outweighs *trans*-regulation in DNA methylation divergence (Figure 2F).

### The causes of *cis*- and *trans*-acting divergence in DNA methylation

To identify the molecular mechanisms driving methylation divergence, we analyzed TFs associated with *trans*-DMRs and sequence variants associated with *cis-*DMRs.

**Figure 3.**
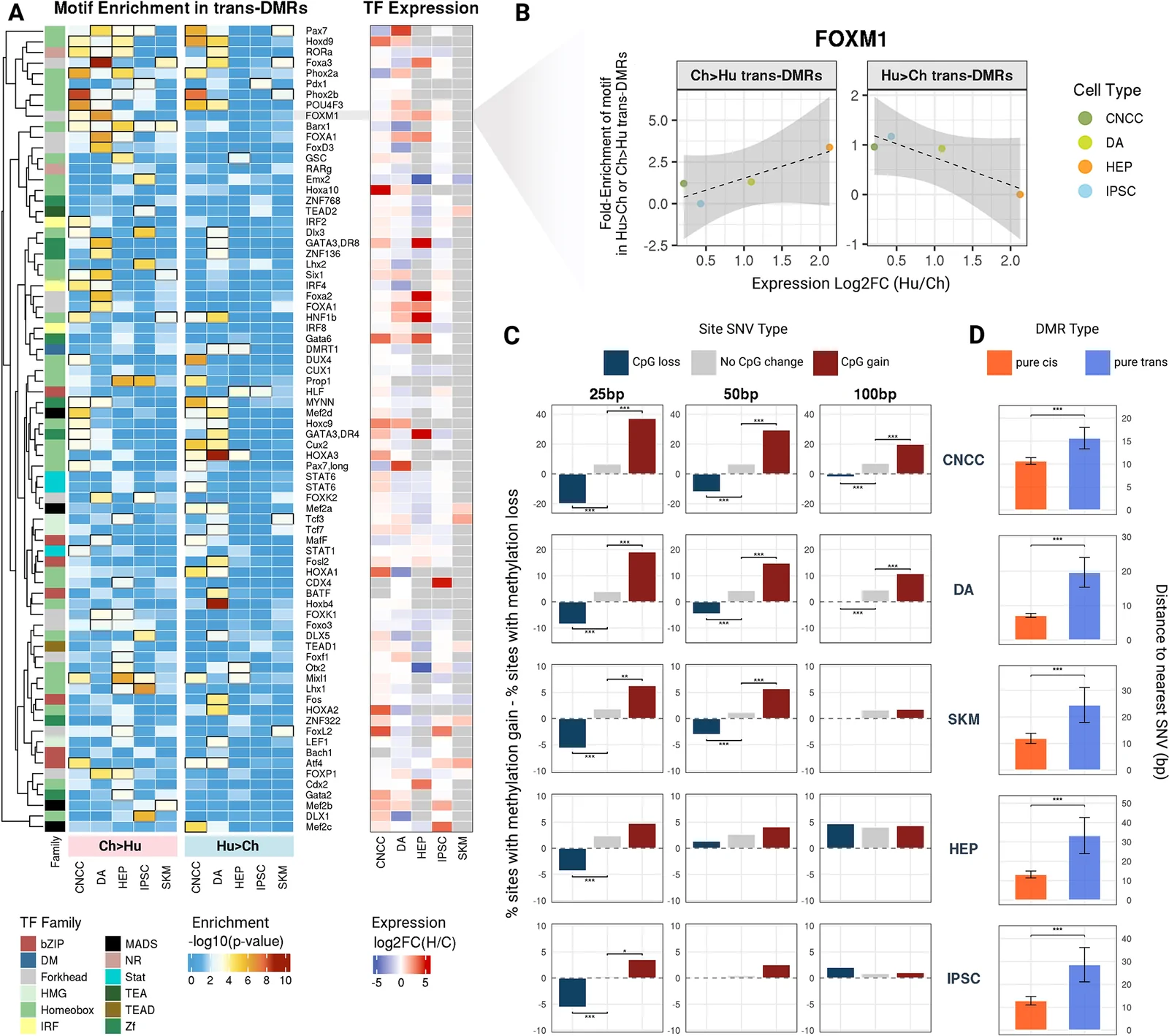
*Cis*- and *trans*-factors influencing species-specific methylation patterns. (**A**) HOMER motif enrichment of *trans-*DMRs (differentially methylated regions) in regions with Hu>Ch and Ch>Hu methylation (left panel), and differential expression of corresponding transcription factors in parents (right panel). (**B**) Fold-enrichments against genomic background of FOXM1, one of the top motifs enriched in Hu>Ch and Ch>Hu *trans-*DMRs, plotted against Hu and Ch relative gene expression levels in parents. (**C**) Three classes of single nucleotide variants (SNVs)—CpG gains in humans, CpG losses in humans, or no CpG change—and their effects on methylation of conserved neighboring CpG sites within ±25, 50, and 100 bp. The differential methylation patterns around CpG SNVs vs. non-CpG-SNVs are assessed using two-proportion test (***: p<0.001, **: p<0.01, *: p<0.05). (**D**) Distances of each *cis-*DMR vs. *trans-*DMR to the nearest CpG SNV site (Mann-Whitney U test; ***: p<0.001).

TFs represent a major class of *trans*-acting factors capable of establishing and maintaining methylation patterns across the genome, often by recruiting methyltransferase or demethylase enzymes. Interspecies variation in TF expression levels, binding activity, or target site accessibility can propagate to downstream methylation differences at their binding sites. To identify TFs potentially driving species-specific methylation divergence through such *trans*-acting mechanisms, we performed TF binding site motif enrichment analysis on *trans*-DMRs (Figure 3A, Supplementary files 4 and 5; Heinz et al., 2010). This analysis identified several candidate factors, including a pioneer factor FOXA2 (Benjamini-Hochberg-corrected FDR = 3.7 × 10^–5^ in DA Hu<Ch *trans*-DMRs) and a chromatin modifier FOXM1 (FDR = 0.002, 4.8×10^–6^, and 0.021 in CNCC, DA, and HEP Hu<Ch *trans*-DMRs, respectively), both known to actively reshape DNA methylation landscapes (Reizel et al., 2021; Li et al., 2022; Gosalia et al., 2015; Iwafuchi-Doi et al., 2016; Lee et al., 2019; Sérandour et al., 2011; Teh et al., 2012).

**Figure 3—figure supplement 1.**
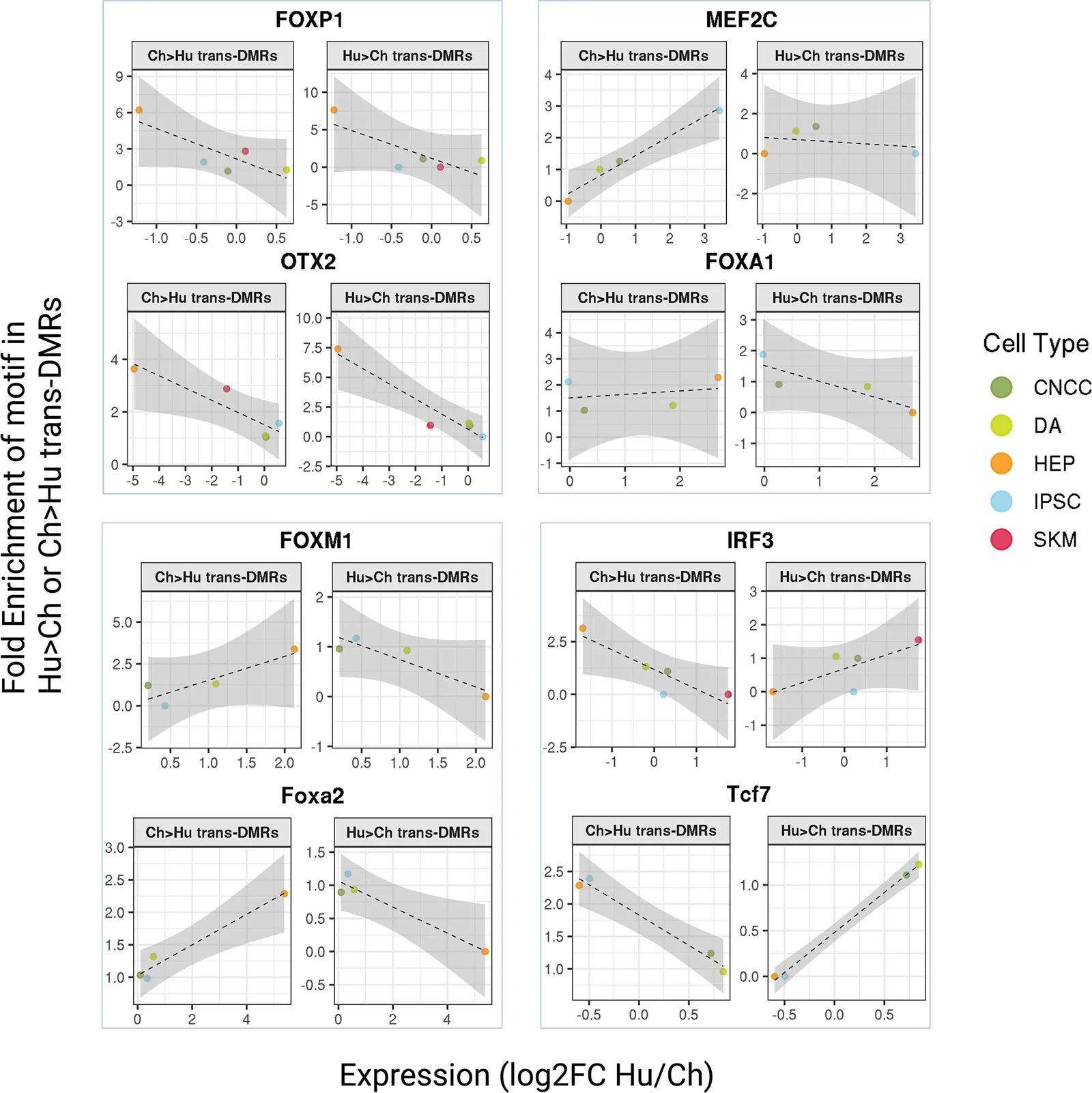
*Trans*-DMR-enriched motifs show different patterns of enrichment relative to differential transcription factor (TF) expression. Fold-enrichments of motifs in Hu>Ch and Hu<Ch *trans*-DMRs are plotted against differential expression of the matching TF in human vs. chimpanzee parental samples. FOXP1 and OTX2 represent cases where there is no consistent directionality in whether *trans*-DMRs are human- or chimpanzee-biased when their differential expression increases. MEF2C, FOXA1, FOXM1, and Foxa2 represent cases where higher expression in a species corresponds to enrichment of the motif in regions where methylation is lower in that species, with FOXM1 and Foxa2 showing a stronger pattern. FOXM1 in this figure is the same as Figure 3B. IRF3 and TCF7 represent cases where higher expression in a species corresponds to enrichment of the motif in regions where methylation is higher in that species. See Supplementary file 4 for detailed statistics.

**Figure 3—figure supplement 2.**
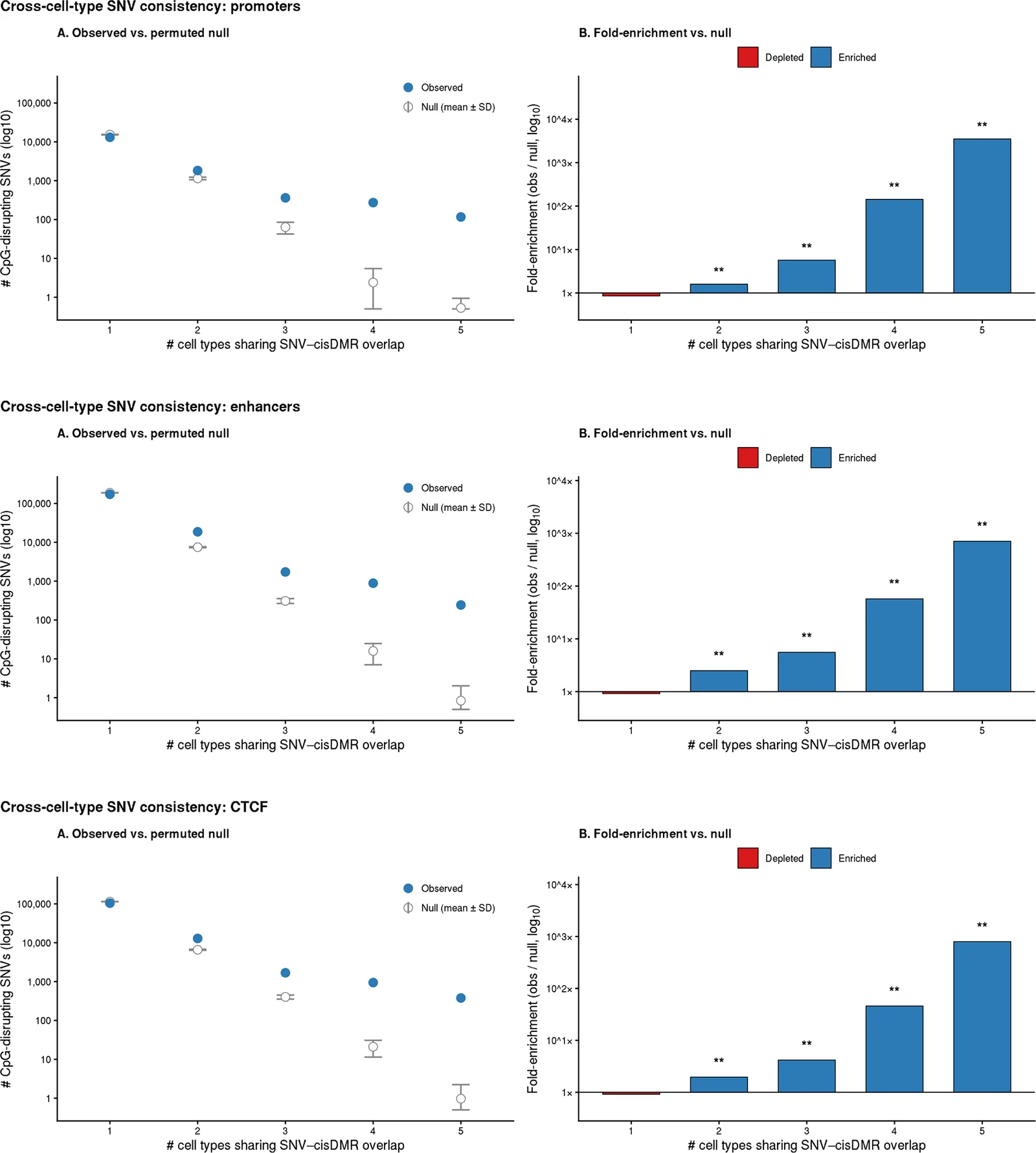
Local methylation effects of CpG single nucleotide variants (SNVs) are shared across cell types. For each ENCODE-annotated regulatory region class (promoters, enhancers, CTCF binding sites), CpG SNVs (±100 bp window) were intersected with *cis*-classified DMRs (‘pure *cis*’ or ‘*cis+trans*’, BH-corrected false discovery rate [FDR]<0.05) across five cell types (cranial neural crest cells [CNCCs], dopaminergic neurons [DAs], hepatocytes [HEPs], induced pluripotent stem cells [iPSCs], skeletal myocytes [SKMs]). For each SNV falling within the tested universe (the union of all assayed regions across cell types), we counted the number of cell types in which it overlapped a *cis*-DMR. (**A**) Number of CpG SNVs (log_10_ scale) sharing *cis*-DMR overlap across one to five cell types. Blue filled points: observed counts; open gray points with error bars: mean ± SD across 1000 permutations of a null model in which, for each cell type, the same number of regions were randomly sampled from that cell type’s pool of tested-but-conserved regions of the same class. (**B**) Fold-enrichment of observed over permuted-null counts (log_10_ scale), with raw fold-change values printed above each bar. Blue bars: enrichment (observed>null); red bars: depletion (observed<null). Asterisks indicate two-sided p-values: *p<0.05, **p<0.01, ***p<0.001 (minimum achievable p=1/1001 ≈ 0.001 with 1000 permutations). CpG SNVs were shared across cell types substantially more than expected by chance for all three region classes (mean cell types sharing per SNV-in-*cis*-DMR: promoters 1.244 vs. null 1.077±0.006; enhancers 1.133 vs. 1.042±0.001; CTCF 1.171 vs. 1.061±0.002; all p<0.001), with fold-enrichment scaling steeply with the degree of sharing (up to ~3500× at promoters, ~800× at CTCF, and ~700× at enhancers for SNVs shared across all five cell types). Singleton SNVs (*cis*-DMR overlap in only one cell type) were correspondingly depleted relative to the null.

If species-specific levels of a TF are responsible for the enrichment of its predicted binding sites in *trans-*DMRs, then this enrichment may be strongest in cell types with greater species-specific differential expression of the TF. FOXM1 and FOXA2 each showed a pattern of this kind, with higher relative expression in one species accompanied by lower methylation at their predicted binding sites in that same species and cell type. FOXM1 was over fourfold more highly expressed in human HEPs than in chimpanzee, and its predicted binding sites were highly enriched specifically for *trans*-DMRs with lower methylation in human HEPs; the three other cell types, in which human and chimpanzee expression of FOXM1 is more similar, showed weaker enrichment and little species-specific asymmetry in *trans*-DMR methylation directionality (Figure 3B). We observed a similar pattern for FOXA2 (Figure 3—figure supplement 1). We note that with only four cell types available per comparison, we are not adequately powered to test for a statistically significant relationship between differential TF expression and motif enrichment. We therefore present FOXM1 and FOXA2 as exploratory, hypothesis-generating examples rather than as evidence of a genome-wide pattern.

Other TFs showed different patterns of association between species-specific expression and *trans*-DMR methylation. For example, FOXP1 and OTX2 were most differentially expressed in HEPs, and their predicted binding sites were also most enriched in *trans*-DMRs in this same cell type (Figure 3—figure supplement 1). However, unlike FOXM1 and FOXA2, binding sites for FOXP1 and OTX2 showed similar patterns of enrichment across cell types for both Hu>Ch and Ch>Hu methylated *trans-*DMRs. This lack of directionality may suggest that these TFs are not restricted to either demethylation or methylation, perhaps having a more indirect or context-specific role in regulating the epigenetic landscape (Braccioli et al., 2018; Lalmansingh et al., 2012; Ee et al., 2025), though further work is required to test this hypothesis. Altogether, our *trans*-DMR analysis nominated many TFs that may contribute to methylation divergence, including several with *trans*-DMR binding site motif enrichment associated with the TF’s differential expression that we highlight for future experimental testing.

To identify the molecular mechanisms driving *cis*-regulatory methylation differences, we then examined whether local sequence variants could explain *cis*-acting methylation divergence. CpG dinucleotides are the primary targets of DNA methylation in mammalian genomes, and their presence, density, and spacing are known to influence local methylation patterns (Hellman and Chess, 2010; Gertz et al., 2011; Zhi et al., 2013). Therefore, SNVs that either create or disrupt CpG dinucleotides represent a plausible mechanism by which sequence divergence could drive species-specific methylation differences. To investigate this hypothesis, we systematically analyzed SNVs and their associated methylation differences in neighboring conserved CpGs within windows of 25, 50, and 100 bp upstream and downstream of each SNV. Our analysis revealed that SNVs affecting CpG dinucleotides—but not other SNVs—are associated with altered methylation patterns of nearby CpG sites up to 50 bp from the variant site (Figure 3C). Notably, the effect diminished with increasing distance from the SNV, with the strongest methylation changes observed within 25 bp and progressively weaker effects at 50 bp. Beyond 50 bp, the magnitude of influence of CpG SNVs on neighboring methylation patterns became negligible, suggesting a limited spatial range for these local sequence effects. This distance-dependent relationship indicates that sequence variants affecting CpG density and spacing might influence the local recruitment or activity of DNA methyltransferases or demethylases. We note that CpG SNVs represent just one of many possible mechanisms for *cis*-methylation divergence; other classes of variants, such as those disrupting TF binding sites, may also contribute and warrant future investigation.

This model predicts that while *cis*-DMRs should often be close to CpG SNVs, *trans*-DMRs—which arise from regulatory mechanisms independent of nearby sequence variation—should not be. To test this, we compared the genomic distance between each DMR and its nearest CpG SNV. Consistent with our model, we found consistently shorter distances for *cis-*DMRs than *trans-*DMRs across all cell types examined: median distances to the nearest CpG SNV were 24±6 bp for *trans*-DMRs vs. 11±2 bp for *cis*-DMRs (Figure 3D; Mann-Whitney p<0.001 for all comparisons) (Mann and Whitney, 1947). This difference suggests that *cis*-DMRs tend to occur near CpG sequence variants and are likely directly influenced by local changes in CpG content, while *trans*-DMRs show no preferential association with CpG variants, as expected given that *trans*-DMRs are by definition not driven by local sequence variation.

To further test the effects of CpG SNVs on local methylation, we assessed whether the same SNVs show consistent methylation effects across multiple cell types. CpG SNVs associated with a *cis*-DMR in one cell type were significantly more likely to be associated with a *cis*-DMR in other cell types than expected by chance, consistent with a shared mechanistic basis for *cis*-methylation divergence across cell types (Figure 3—figure supplement 2). This genomic organization is consistent with *trans-*DMRs being regulated by diffusible factors such as TFs rather than by local sequence variants, supporting a model in which *cis*- and *trans*-acting mechanisms operate through fundamentally different molecular pathways to generate species-specific methylation patterns.

### Cell-type- and species-specific gene expression as a consequence of methylation divergence

**Figure 4.**
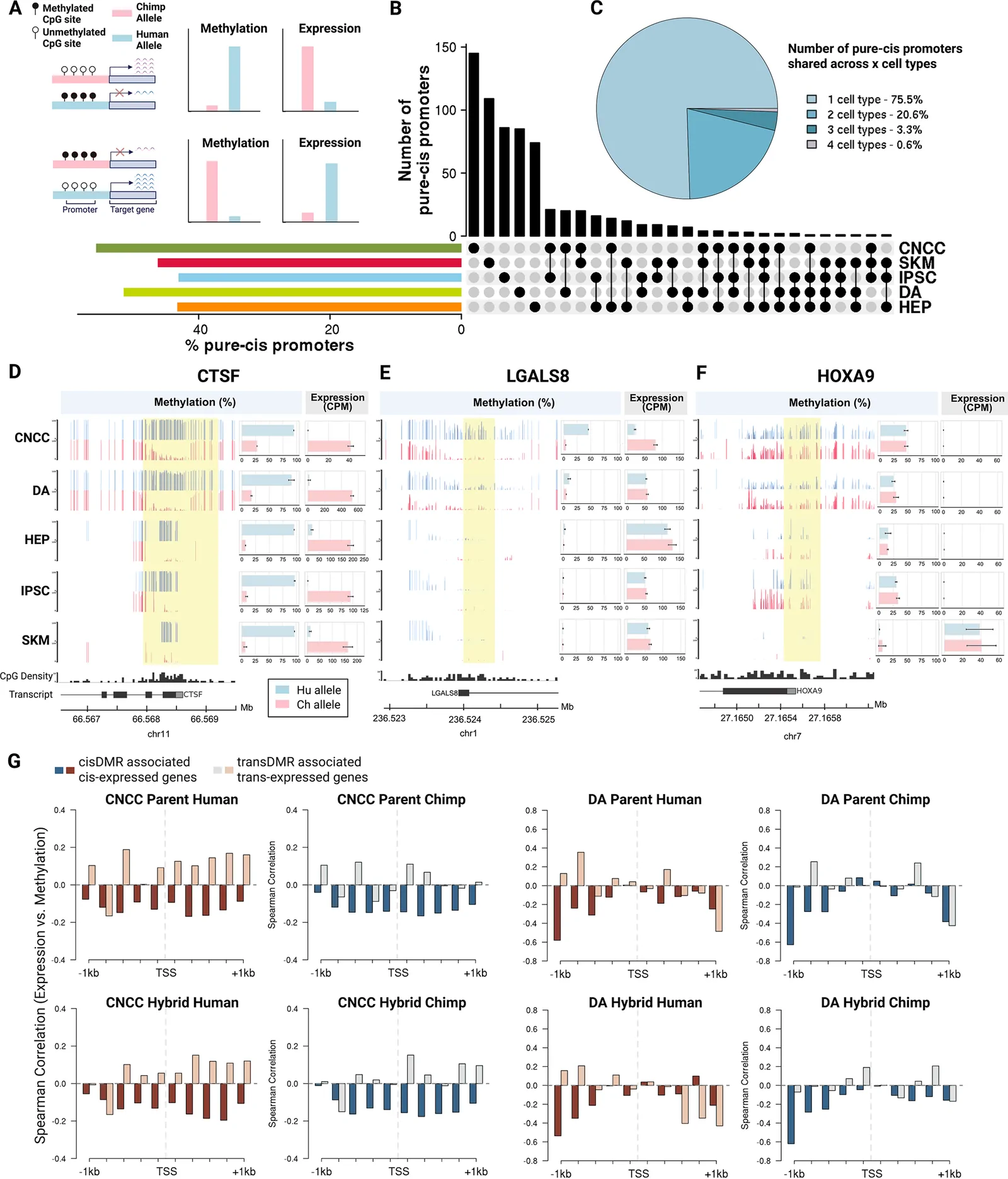
Human-chimpanzee allele-specific methylation can be associated with allele-specific expression. (**A**) Illustration of the transcriptional consequences of DNA methylation explored in this study. DNA methylation, as a stable *cis*-acting regulatory mark, often represses transcription. In a hybrid, allele-specific methylation can lead to allele-specific gene expression, although both methylation and its effects on transcription can be context-dependent. (**B**) We identified many promoters with allele-specific methylation in each cell type, most of which showed allele-specific methylation in only one cell type. (**C**) Quantification of promoters with pure-*cis*-regulation of methylation across cell types. (**D–F**) Examples of gene expression-methylation correspondence. Methylation tracks show fractional methylation (mC/coverage × 100%) of individual CpGs. Expression values are in TMM (trimmed mean of M-values)-normalized CPM (counts per million). (**D**) *CTSF* as an example of species-specific, cell-type-agnostic DMR, (**E**) *LGALS8* as an example of cell-type-specific allele-specific methylation, and (**F**) *HOXA9* as an example of cell-type-specific but species-agnostic methylation patterns. (**G**) Genome-wide profile of expression-methylation correlations (binned by distance of methylated region to the transcription start site [TSS]) for genes with both promoter methylation and expression under *cis*-regulation (dark red and dark blue) or *trans*-regulation (light brown and light gray). Detailed statistics are provided in Supplementary file 6. Created with BioRender.com.

Given the well-established repressive effect of promoter methylation on gene expression, we assessed whether methylation differences contribute to expression divergence by analyzing genome-wide correlations between allele-specific promoter methylation and expression levels across different genomic contexts (Figure 4A; Jaenisch and Bird, 2003; Murrell et al., 2005; Stein et al., 1982). We first examined whether *cis*-acting variants drive promoter methylation consistently across cell types or in a context-dependent manner. We found that while some promoters are allele-specifically methylated across all cell types, many more showed cell-type-specific *cis*-methylation patterns, suggesting that promoter methylation regulation is often context-dependent (Figure 4B and C). However, while DMRs consistently detected across cell types provide strong evidence of shared regulation, DMRs detected in only one cell type may reflect either genuine cell type specificity or insufficient power to detect them elsewhere. Our estimate of cell-type-specific regulation therefore represents an upper bound on the degree of cell-type-specific methylation regulation.

**Figure 4—figure supplement 1.**
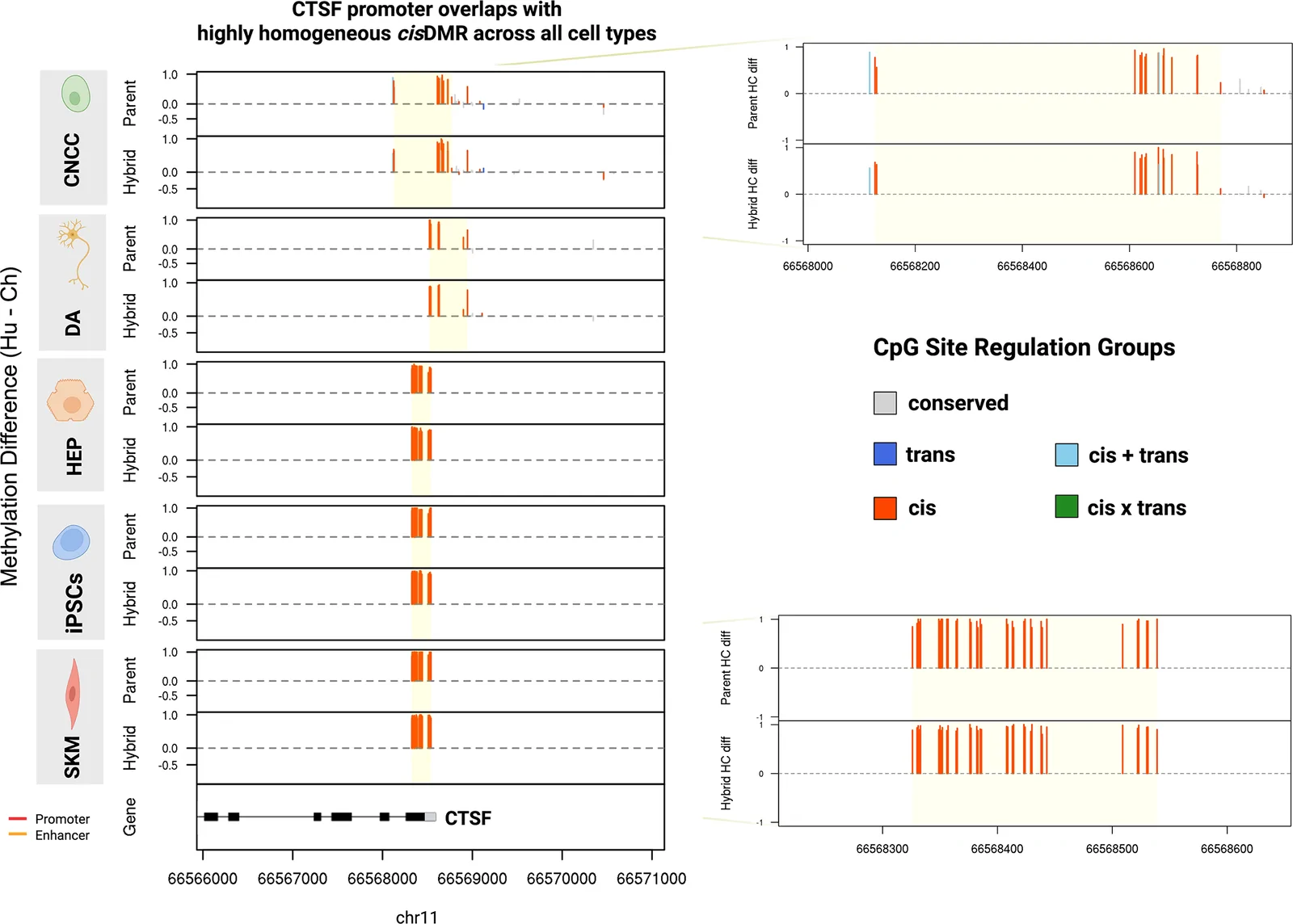
*CTSF* promoter overlaps with highly homogeneous *cis*-DMR (differentially methylated region) across all cell types. Differences of fractional methylation between human and chimpanzee in hybrid and parental cell types are compared. Each vertical line represents methylation difference (Hu-Ch) of a single CpG site, colored by its regulation groups informed by its allele-specific and differential methylation levels. Consistent across all cell types, a homogeneous cluster of *cis*-regulated CpG sites identified as *cis*-DMR overlaps with the *CTSF* promoter, meaning that besides the entire promoter region being classified as a *cis*-DMR promoter using pooled methylation counts, the majority of individual CpG sites within the promoter region are individually classified as *cis* CpG sites. See Supplementary file 3 for detailed statistics.

**Figure 4—figure supplement 2.**
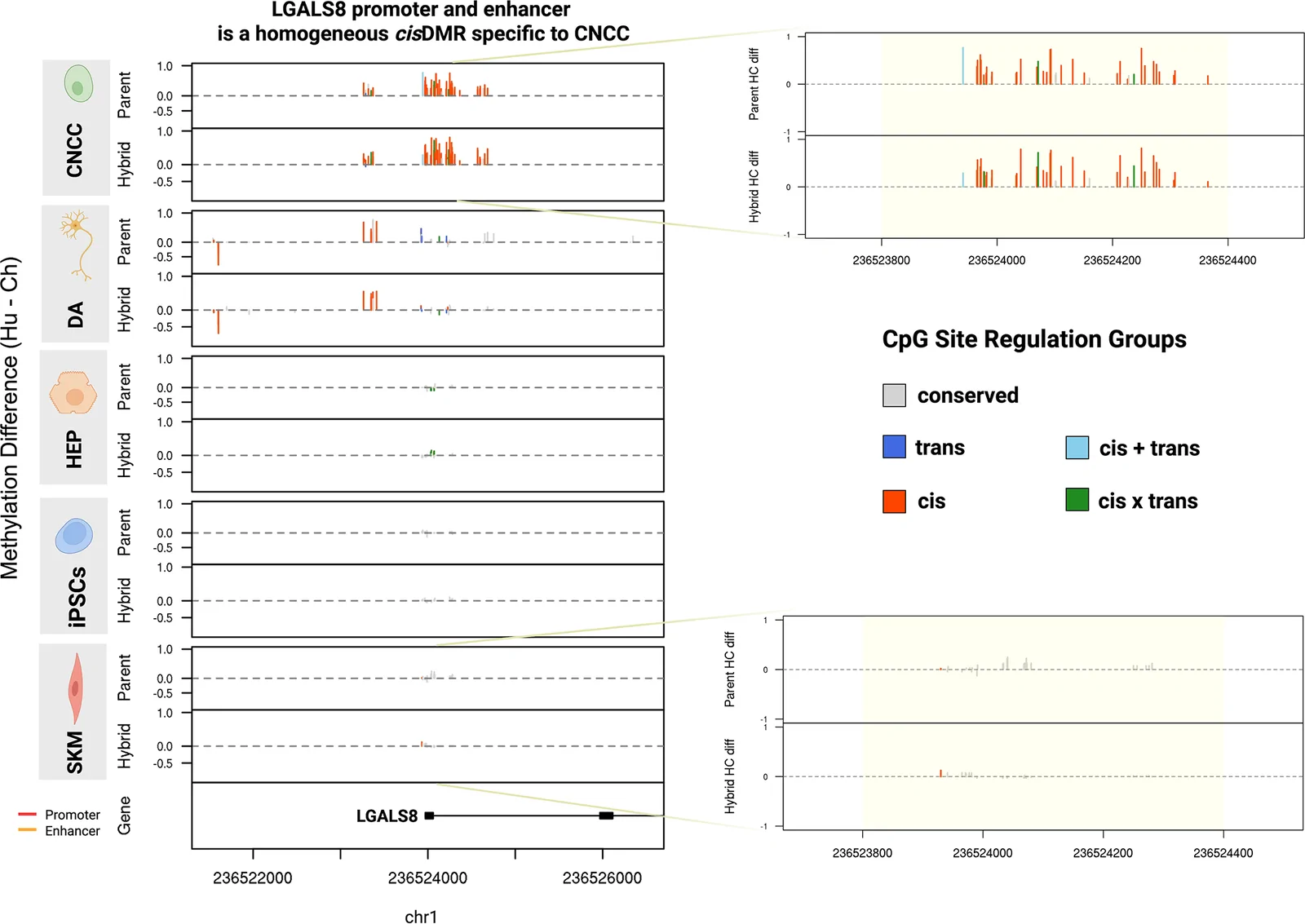
*LGALS8* promoter and enhancer is a homogeneous *cis*-DMR (differentially methylated region) specific to cranial neural crest cell (CNCC). Differences of fractional methylation of human and chimpanzee in hybrid and parental cell types are compared. Each vertical line represents methylation difference of a single CpG site, colored by its regulation group. From Figure 4E, it is evident that the *LGALS8* promoter and enhancer region shows allele-specific methylation exclusively in CNCC while showing no allelic differences in other cell types. As seen from this figure, the allele-specific methylated region is also differentially methylated in CNCC parental cells and the majority of individual CpG sites are characterized as *cis*, meaning that this region is a *cis*-DMR, whereas other cell types show conserved methylation in the same region. See Supplementary file 3 for detailed statistics.

**Figure 4—figure supplement 3.**
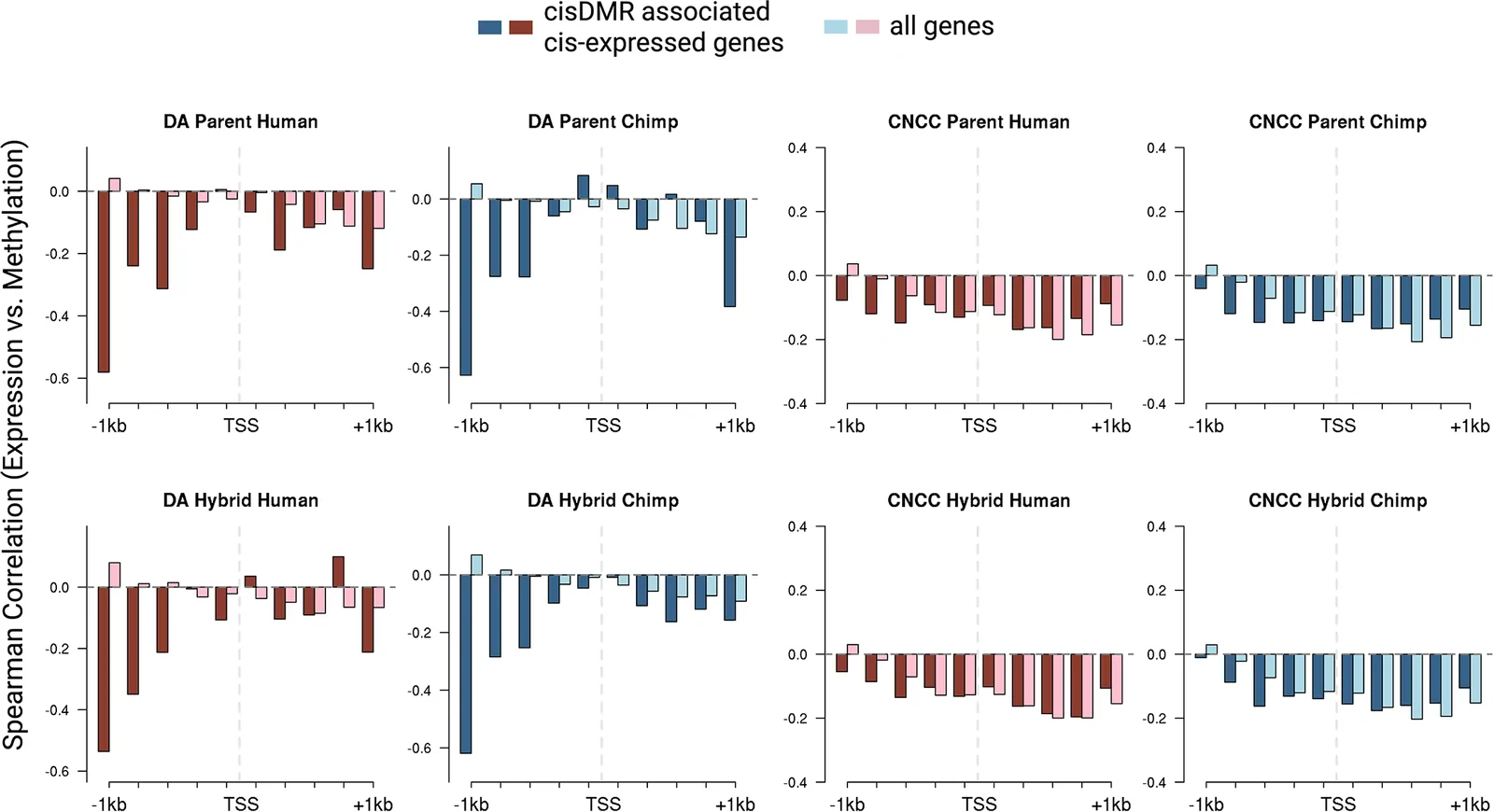
Spearman correlation of expression and methylation for cranial neural crest cell (CNCC) and dopaminergic neuron (DA). Binned expression-methylation correlation profile of genes with both methylation and expression under *cis*-regulation (dark red and steel blue) and all genes regardless of regulation type (light pink and light blue). Two kb surrounding each transcription start site (TSS) is shown, split into 10 bins of 200 bp where individual CpG sites are pooled and averaged as fractional methylation values and correlated with expression values of the neighboring genes (in transcript per million [TPM]). A weak but significant negative correlation is seen between expression and methylation levels near the promoter regions slightly upstream of TSS, consistent with past studies. When restricting to only the genes with *cis*-regulated methylation and *cis*-regulated expression, the correlation is stronger, especially in the bin 800 bp to 1 kb upstream of the TSS in DA cells. See Supplementary files 5 and 6 for significance and detailed statistics.

**Figure 4—figure supplement 4.**
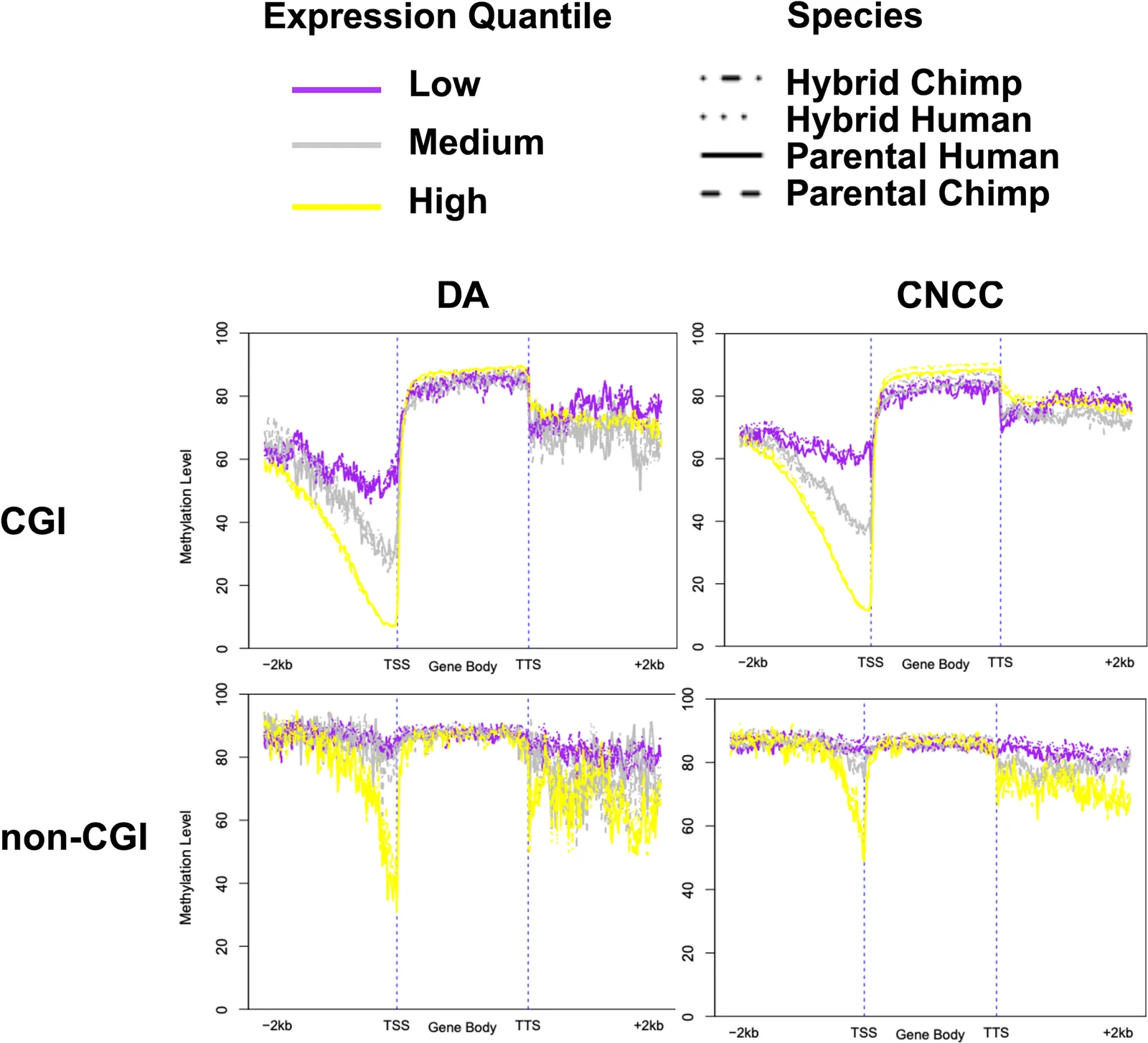
CpG island (CGI) promoters show stronger repressive methylation-expression coupling than non-CGI promoters. Genes were stratified into three equally sized groups based on their expression levels (transcript per million [TPM]), and average methylation level of each group is shown in 100 bins across 2000 bp upstream of transcription start site (TSS), gene body (all gene bodies scaled to be equal length in this plot), as well as 2000 bp downstream of transcription termination site (TTS). A general pattern follows for CGI promoters that high expression is associated with low methylation, medium expression is associated with intermediate methylation levels, and low expression is associated with high methylation, whereas non-CGI promoters show less stratification. Analysis of both CGI and non-CGI promoters revealed that genes are generally hypomethylated in promoter regions, with highly expressed genes showing a higher degree of hypomethylation in promoters, consistent with the established role of promoter methylation in transcriptional repression. However, CGI promoters showed more pronounced stratification, with highly expressed genes displaying significantly lower promoter methylation levels compared to lowly expressed genes.

**Figure 4—figure supplement 5.**
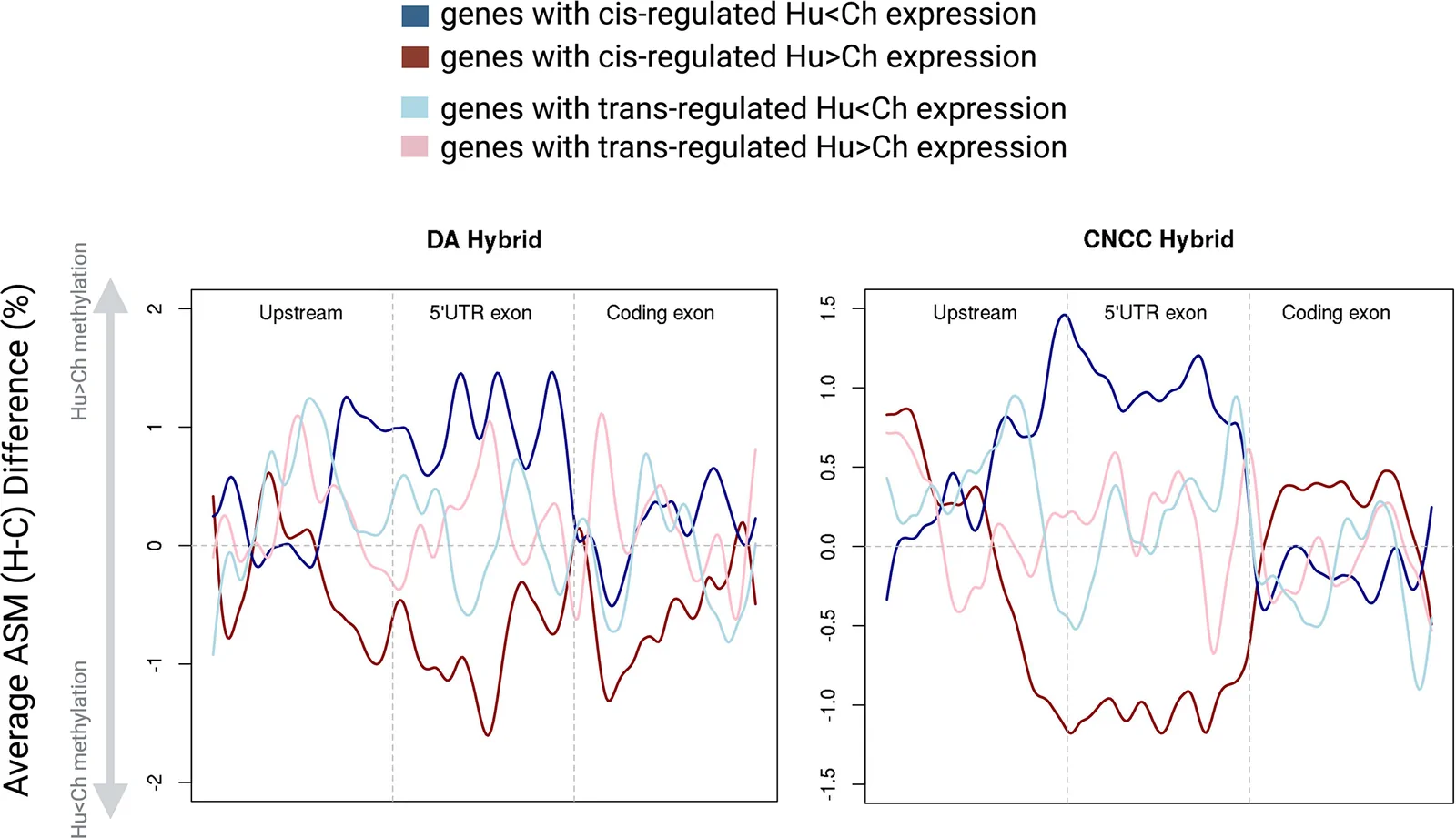
Genes with *cis*-regulated expression are more likely to exhibit repressive patterns (i.e. higher methylation associated with lower expression) than those with *trans*-regulated expression. The upstream regions (1000 bp upstream of TSS), gene bodies, and downstream regions (1000 bp downstream of transcription termination site [TTS]) are each split into 100 equal bins where individual CpG sites are pooled and averaged as fractional methylation values. In genes with *cis*-regulated gene expression, the ones with Hu<Ch expression show Hu>Ch methylation on average across ~1000 bp upstream of and including 5’ UTR region whereas genes with Hu>Ch expression show Hu<Ch methylation. In genes with *trans*-regulated gene expression, the repressive pattern is not observed. This is consistent with DNA methylation acting as an upstream *cis*-regulatory mechanism.

Several specific examples illustrate different patterns of methylation-expression coordination (Supplementary files 5 and 6; see Materials and methods). The promoter of *CTSF* is a cell-type-agnostic *cis*-DMR with consistent Hu>Ch methylation across cell types corresponding to coordinated Ch>Hu expression (Figure 4D, Figure 4—figure supplement 1). In contrast, a putative enhancer in *LGALS8* represents a case where methylation divergence is confined to specific cellular contexts. The candidate enhancer overlaps with a *cis-*DMR with Hu>Ch methylation and *LGALS8* exhibits allele-specific Ch>Hu expression exclusively in CNCCs, while having no divergence in methylation or expression in other cell types (Figure 4E, Figure 4—figure supplement 2). A third type of pattern is exemplified by the *HOXA9* promoter, with cell-type-specific hypomethylation corresponding to cell-type-specific expression only in SKM (Figure 4F). These examples illustrate how different regulatory mechanisms can produce distinct patterns of methylation-expression coordination and how epigenetic changes may contribute to both species-specific and cell-type-specific regulatory evolution.

Previous studies have reported weak but significant correlations between DNA methylation and gene expression divergence in primates (Blake et al., 2020; Pai et al., 2011; Zeng et al., 2012; Hernando-Herraez et al., 2013; Wang et al., 2024a). Consistent with these findings, our genome-wide estimates of human-chimpanzee transcriptome divergence produced similar weak but significant correlations with methylation divergence (Figure 4—figure supplement 3 and Supplementary file 6). Our comparison of CpG island (CGI) vs. non-CGI promoters also yielded trends consistent with past studies showing that CGI promoter methylation correlates more strongly with gene expression repression than does non-CGI promoter methylation (Saxonov et al., 2006; Boman et al., 2024; Figure 4—figure supplement 4).

However, such genome-wide correlations may mask stronger relationships that occur within specific regulatory contexts where methylation and expression are more directly coupled. We hypothesized that our hybrid system could help identify genes exhibiting stronger methylation-expression coupling by stratifying them based on regulatory mechanisms. We reasoned that genes whose methylation and expression are both influenced by *cis*-acting variants—potentially driven by the same underlying genetic differences—should display tighter coupling than genes regulated through independent mechanisms.

To test this, we evaluated the extent to which methylation and expression show a negative relationship (i.e. higher methylation associated with lower expression) consistent with a repressive effect of methylation. In both CNCC and DA hybrid cells, we found that genes with *cis*-regulated expression were more likely to exhibit repressive patterns than those with *trans*-regulated expression (Figure 4—figure supplement 5). This is consistent with DNA methylation acting as an upstream *cis*-regulatory mechanism: genes with stronger *cis*-regulation are expected to show tighter methylation-expression coupling due to the greater contribution of local regulatory factors, including methylation itself. Further restricting the analysis to genes with concordant regulatory mechanisms revealed that genes with both methylation and expression being *cis*-regulated (‘*cis*-*cis*’ genes; Supplementary file 8) exhibited significantly stronger negative methylation-expression correlations than ‘*trans*-*trans*’ genes (Figure 4G, see Supplementary file 6 for detailed statistics). This supports the hypothesis that methylation-expression coupling is strongest when both are driven by local genetic variation, perhaps through shared causal variants. Different shapes of the methylation-expression correlation profiles around the TSS in each cell type may reflect differences in the chromatin landscape and TF occupancy that modulate the functional impact of promoter methylation in each cell-type context; a deeper mechanistic explanation is left for future work.

### The sign test identifies lineage-specific selection on DNA methylation between human and chimpanzee

To identify pathways that have evolved under lineage-specific selection, we used a two-step binomial sign test approach that tests for non-random directional bias in methylation-expression patterns in hybrids (Fraser, 2013; Agoglia et al., 2021; Gokhman et al., 2021; Wang et al., 2024b; Fraser, 2011; Sharon et al., 2018; Artieri and Fraser, 2014; Nourmohammad et al., 2017; Papakostas et al., 2014). The use of hybrid cell lines is critical for this analysis as it ensures that different promoters represent independent observations, controlling for *trans*-acting factors and environmental variation that could otherwise confound the detection of *cis*-regulatory effects. This test operates on the logic that neutral evolution should act like flipping a fair coin: if human-biased and chimpanzee-biased changes occur at equal background frequencies, then methylation and expression changes of genes within a pathway would accumulate randomly, showing no preference for human-biased vs. chimpanzee-biased directionality. In contrast, lineage-specific selection would bias the coin flip, potentially causing functionally related gene sets to consistently favor one direction over the other rather than splitting evenly by chance.

To maximize our ability to find phenotypically impactful divergence, we adapted the standard sign test to account for directionality of both methylation and gene expression. In the first step, we applied a standard binomial sign test to all 6283 promoters showing ASM, counting the number of genes showing human-biased vs. chimpanzee-biased methylation changes within each gene set. The background probability for this test was defined as the overall fraction of promoter ASM in each cell type that was human-biased. This initial analysis identified gene sets showing significant enrichment for directional methylation changes, establishing candidates for lineage-specific regulatory divergence. In the second step, we restricted our attention to genes showing a negative association between ASM and ASE. This subset represents cases where methylation changes are most likely to have a simple and direct repressive effect on transcription, filtering out more complex regulatory scenarios where methylation and expression changes may be uncoupled or subject to competing regulatory influences.

**Figure 5.**
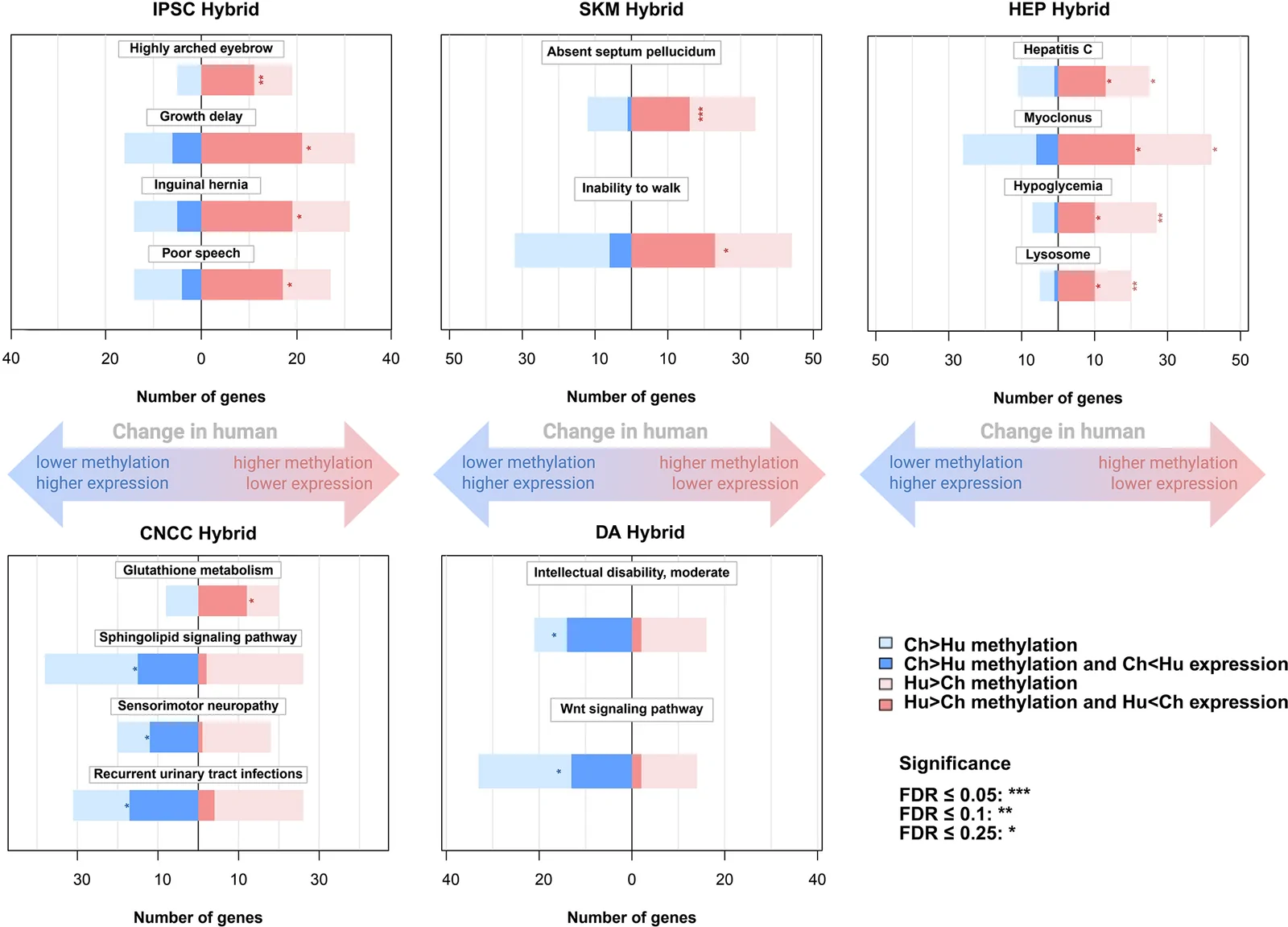
Gene sets with evidence of lineage-specific selection on methylation and gene expression. The length of the bars indicates the number of genes in each gene set with allele-specific expression or methylation in each direction (human- or chimpanzee-biased). The light bars show all genes in each gene set with human- or chimpanzee-biased allele-specific methylation; the dark bars show the subset of genes whose allele-specific expression is in the direction expected from repressive methylation (higher methylation, lower gene expression).

Gene sets showing significant deviation from neutral expectation in the ASM/ASE concordance test (nominal binomial p-value<0.05 and FDR<0.25 after multiple test correction; see Materials and methods), with supporting evidence of directional bias in the methylation-only sign test (nominal binomial p-value<0.05), are interpreted as likely targets of lineage-specific selection on promoter methylation that could directly impact gene expression (Figure 5, Supplementary file 7). However, as indicated by the FDR, we do expect some fraction of these candidate gene sets to be false positives. Compared to a standard sign test that focuses solely on directionally biased gene expression (Fraser, 2011), this more stringent two-step filtering approach may identify higher-confidence candidates where regulatory divergence is reflected at both the methylation and expression levels, establishing a potential mechanistic connection to phenotype.

**Figure 5—figure supplement 1.**
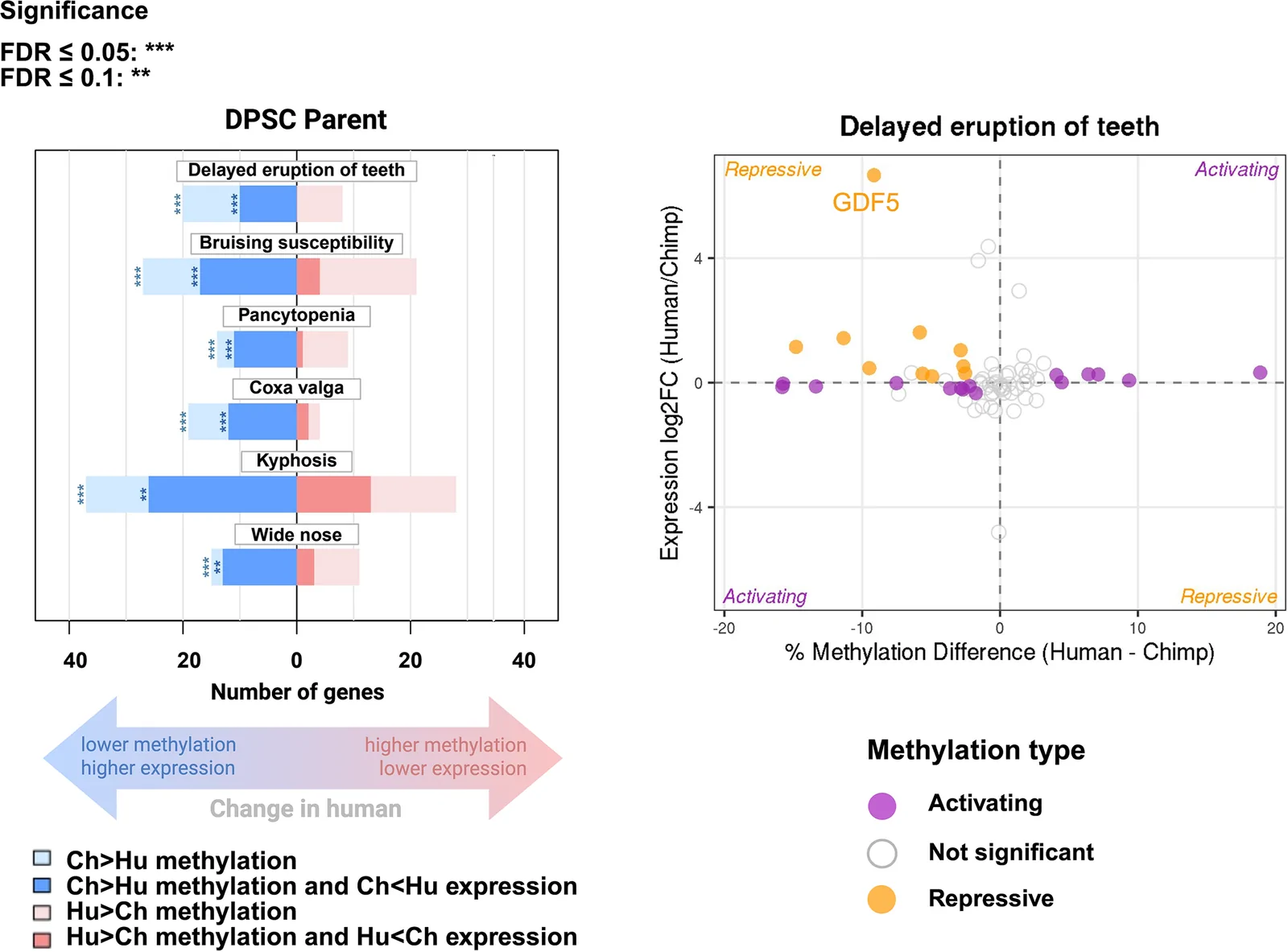
Sign test on dental pulp stem cell (DPSC) parental methylation and expression data shows significant bias for genes related to delayed eruption of teeth. Left panel: The length of the bars indicates the number of genes in a category with allele-specific expression or methylation in each direction (human- or chimpanzee-biased). The bars in bolded colors represent the number of genes within the pathway where higher methylation is associated with repressed gene expression, whereas lighter colors represent genes where higher methylation is associated with increased gene expression. Right panel: Differential expression log fold-changes are plotted against methylation. Notably, in pathway ‘delayed eruption of teeth’, *GDF5* has >30-fold higher expression and ~10% lower promoter methylation in human compared to chimpanzee DPSCs. Since the sign test assumes independence of gene expression changes within a pathway, results on parental data (which do not exclude *trans*-acting factors that may be shared across multiple genes) are suggestive but cannot reject the null hypothesis of neutral evolution. DPSC parental samples had a strong genome-wide human bias in promoter methylation, with 68% of promoters showing human-biased methylation compared to 32% chimp-biased, increasing the significance of gene sets with chimp-biased methylation. See Supplementary file 7 for detailed statistics.

**Figure 6.**
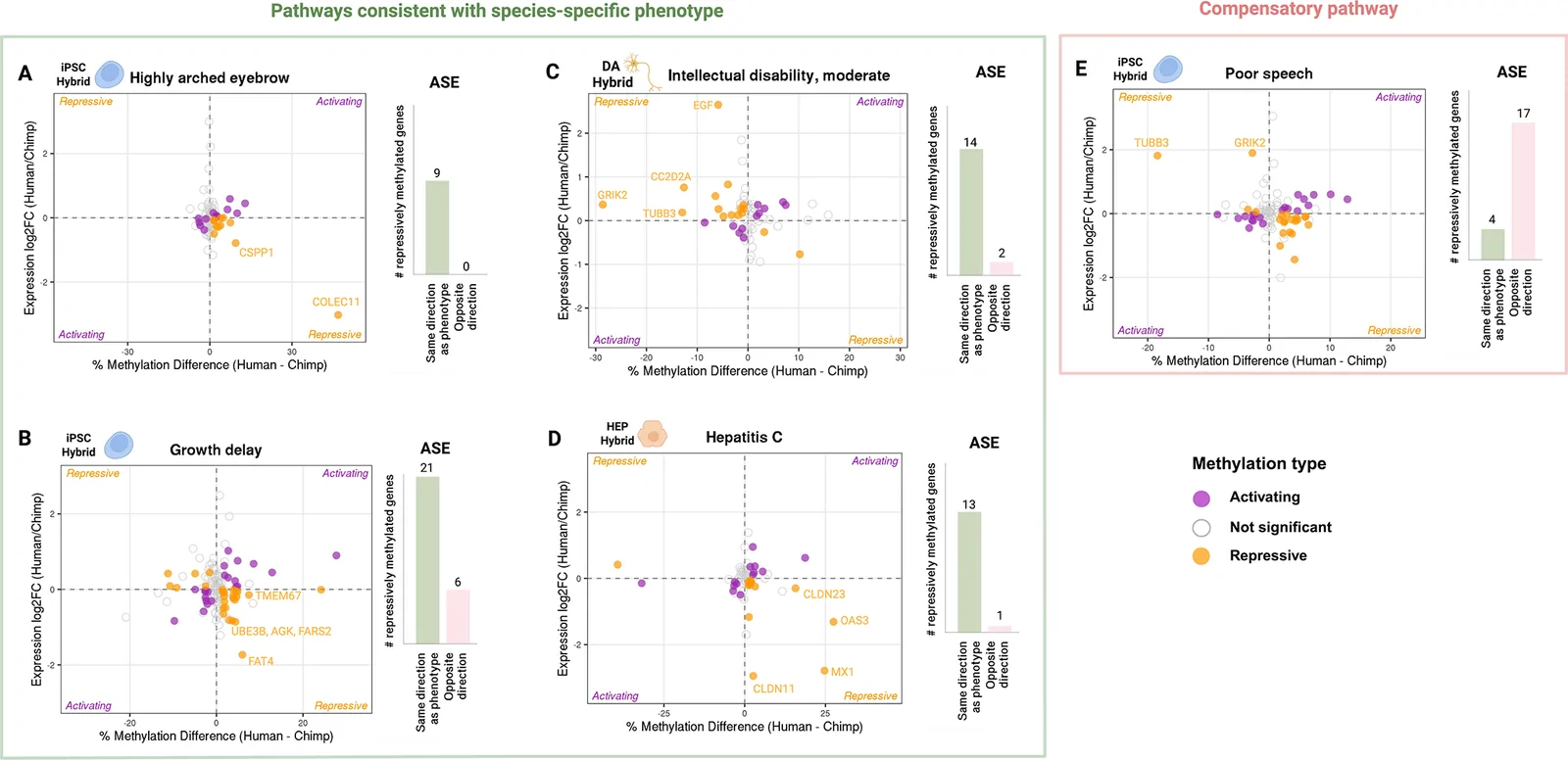
Directional bias in allele-specific methylation (ASM) and gene expression identifies candidate genes for human-specific phenotypes. Allele-specific expression (ASE) is plotted against ASM. Orange dots represent genes consistent with repressive methylation (e.g. Hu>Ch ASE and Hu<Ch ASM) and purple dots represent genes consistent with activating methylation (e.g. Hu>Ch ASE and Hu>Ch ASM). Bar plots represent counts of genes with repressive methylation that exhibit expression changes in directions either consistent with (light green) or opposite to (light pink) what would be expected based on phenotypic differences between humans and chimpanzees. (**A**) The ‘highly arched eyebrow’ gene set in induced pluripotent stem cell (iPSC) hybrids shows consistent Hu<Ch ASE and Hu>Ch ASM for genes with repressive methylation. (**B**) The ‘growth delay’ gene set in iPSC hybrids shows significant bias toward Hu<Ch ASE and Hu>Ch ASM for genes with repressive methylation. (**C**) The ‘intellectual disability, moderate’ gene set in dopaminergic neuron (DA) shows consistent Hu>Ch ASE and Hu<Ch ASM for genes with repressive methylation, with genes including *GRIK2, TUBB3, EGF*, and *CC2D2A* being among the genes with highest magnitude ASE and ASM. (**D**) The ‘hepatitis C’ gene set in hepatocyte progenitor (HEP) shows consistent Hu<Ch ASE and Hu>Ch ASM for genes with repressive methylation. (**E**) The ‘poor speech’ gene set in iPSC hybrids represents a potentially compensatory pathway where most genes with repressive methylation show Hu<Ch ASE and Hu>Ch ASM, whereas the genes with highest magnitude of ASE and ASM (*TUBB3* and *GRIK2*) show Hu>Ch ASE and Hu<Ch ASM.

Among gene sets from Kyoto Encyclopedia of Genes and Genomes (KEGG) and Human Phenotype Ontology (HPO) (Kanehisa and Goto, 2000; Gargano et al., 2024), we identified several with clear relevance to human-specific traits. In iPSC hybrids, multiple gene sets including ‘poor speech’, ‘highly arched eyebrow’, and ‘growth delay’ all showed Hu>Ch methylation and Ch>Hu expression (Figure 5 and Figure 6A, B). In the SKM hybrids, genes related to ‘inability to walk’ showed significant Hu>Ch methylation and Hu<Ch expression (Figure 5). In DA hybrids, genes involved in ‘intellectual disability, moderate’ show significant Hu<Ch methylation and Hu>Ch expression (Figures 5 and 6C). In HEP hybrids, we identified the ‘hepatitis C’ gene set with significant Hu>Ch methylation and Hu<Ch expression (Figures 5 and 6D). In primary DPSC samples, we identified significant bias toward Hu<Ch methylation and Hu >Ch expression in genes related to tooth eruption timing, consistent with known differences in dental development between humans and chimpanzees, where human permanent molars emerge approximately 6 years apart compared to 3–4 years in chimpanzees (Figure 5—figure supplement 1; Dean, 2006; Dixon et al., 2017; Smith et al., 2013). However, the DPSC sign test results should not be interpreted as evidence of lineage-specific selection due to the use of parental rather than hybrid cell lines, which may introduce dependencies among observations due to *trans*-acting effects, potentially inflating statistical significance. For all other cell types (iPSCs, DAs, SKMs, HEPs, CNCCs), the sign test results are based on hybrid lines and can be interpreted as evidence of lineage-specific selection, as the hybrid system controls for *trans*-acting confounders.

We then analyzed individual genes within each candidate gene set to assess whether expression bias in associated phenotypes aligns with known human-chimpanzee trait differences, focusing on genes showing repressive methylation. Multiple gene sets demonstrated directional bias in gene expression consistent with the direction of phenotypic divergence found between humans and chimpanzees. For instance, in the ‘highly arched eyebrow’ gene set—where most genes show human downregulation in iPSC hybrids—*COLEC11* (which has eightfold lower expression from the human allele) stands out as an outlier in magnitude. *COLEC11* acts as a guidance cue in directing neural crest cell migration during early development, and in humans its loss-of-function (LOF) causes craniofacial dysmorphism, including highly arched eyebrows and underdeveloped brow ridge, both of which are traits unique to humans (Figures 5 and 6A; Rooryck et al., 2011). In the ‘growth delay’ pathway, most genes with repressive methylation (whose LOF in humans and other species leads to delayed growth) show lower expression from their human alleles, consistent with humans’ substantially delayed growth pattern compared to chimpanzees (Figure 6B; Jones, 2011; Davison and Gurven, 2021). Many of the genes in DA whose LOF causes ‘intellectual disability, moderate’ may have contributed to the evolution of human cognitive abilities. For example, the high-magnitude genes with Hu>Ch ASE and Hu<Ch ASM such as *GRIK2*, *TUBB3, EGF,* and *CC2D2A* are essential for excitatory synaptic plasticity and long-range axon wiring (Figure 6C; Stolz et al., 2021; Valbuena and Lerma, 2021; Tischfield et al., 2010; Poirier et al., 2010; Romano and Bucci, 2020; Noor et al., 2008). In addition, most genes whose inhibition or LOF increases susceptibility or severity of hepatitis C (e.g. *MX1* and *OAS3*) show lower expression from human alleles in HEP, consistent with humans’ heightened vulnerability to hepatitis C viral infections (Figure 6D; Li et al., 2016; Yee et al., 2007; Walker, 1997).

Interestingly, we also discovered gene sets where the direction of most gene expression differences predicted the opposite of a known human/chimpanzee phenotypic difference. For example, in the ‘poor speech’ gene set, where LOF is associated with impaired speech in humans, we counterintuitively observed that most genes were downregulated in humans compared to chimpanzees. However, the two genes with the largest fold-changes, *TUBB3* and *GRIK2*, showed higher expression from the human allele (Figure 6E). Notably, these two genes are also among the large-magnitude genes in the predominantly human upregulated ‘intellectual disability, moderate’ pathway, which does align with species-specific phenotype (Figure 6C). This suggests the speculative possibility of compensatory evolution in which large, pleiotropic effects of a few genes are counterbalanced by smaller changes in many genes (Figure 6C and E; also see Discussion). Our previous study found that genes affecting the voice box show particularly extensive downregulation in humans, consistent with our observation in the ‘poor speech’ pathway (Gokhman et al., 2021).

Collectively, these results provide the first evidence for lineage-specific selection on DNA methylation. Our use of hybrid cell lines was critical to this discovery, as it allowed us to isolate *cis*-acting regulatory changes from confounding *trans*-acting and environmental effects. Isolating *cis*-acting changes is critical because the sign test requires that each promoter represents an independent observation; in hybrid cells, each allele’s methylation is determined by its own *cis*-regulatory sequence, making observations across promoters genuinely independent and thus satisfying the assumptions of the binomial test. This approach reveals that polygenic selection has acted on entire sets of functionally related genes whose expression is modulated through DNA methylation. Importantly, by connecting lineage-specific methylation divergence to expression changes within functionally coherent pathways, we can generate plausible hypotheses linking regulatory evolution to human-specific phenotypes. These include craniofacial morphology, dental development, viral infection susceptibility, and neurodevelopmental characteristics underlying speech and cognition (Figures 5 and 6, Figure 5—figure supplement 1).

## Discussion

Our investigation using human-chimpanzee hybrid tetraploid cells revealed that *cis*-regulatory mechanisms are the primary drivers of genome-wide methylation divergence between humans and chimpanzees across multiple cell types, including DAs, skeletal muscle, iPSCs, HEPs, and CNCCs. This finding aligns with previous studies highlighting the predominant role of *cis*-regulatory elements in evolutionary adaptations, as they enable precise, context-specific gene regulation while minimizing the potentially deleterious pleiotropic effects of *trans*-acting factors (Wittkopp and Kalay, 2012). We demonstrated that most differentially methylated sites arise through *cis*-regulatory mechanisms, with *trans*-regulation and *cis-trans* interactions playing secondary roles. We identified clusters of CpG sites with consistent regulatory mechanisms, forming coherent DMRs. These DMRs predominantly overlap with functionally relevant genomic features, including 5' UTRs and upstream promoter regions, underscoring their regulatory significance.

Our mechanistic analysis revealed distinct pathways through which *cis*- and *trans*-regulatory factors establish species-specific methylation patterns. For *cis*-regulation, we identified CpG-altering sequence variants as a direct mechanism linking genomic divergence to epigenetic differences. These variants influence methylation patterns of neighboring CpG sites with effects diminishing with genomic distance. Importantly, *cis-*DMR clusters are significantly closer to CpG SNVs than *trans-*DMRs across all cell types, providing evidence that local sequence variation directly shapes *cis*-regulatory methylation patterns, perhaps through altered CpG density affecting recruitment of DNA methyltransferases or demethylases.

Our analysis of allele-specifically methylated promoters revealed that the majority showed cell-type-specific patterns. Our examples illustrate this diversity: the *CTSF* promoter exhibits consistent cross-cell-type *cis*-methylation divergence (Figure 4—figure supplement 1) with corresponding expression changes; an enhancer near *LGALS8* shows cell-type-specific ASM and overlaps with a homogeneous *cis-*DMR exclusively in CNCCs; and the *HOXA9* promoter displays cell-type-specific methylation patterns with corresponding context-dependent expression effects. These findings highlight how the broader regulatory context of each cell-type impacts the effects of *cis*-regulatory variants.

Our genome-wide correlation analyses confirmed the well-known pattern that while promoter methylation generally shows statistically significant inverse relationships with gene expression, these correlations are weak when examined across all genes. However, by leveraging our hybrid cell system to stratify genes based on regulatory mechanism, we uncovered significantly stronger negative correlations between methylation and expression when both are *cis*-regulated compared to genome-wide estimates or *trans*-regulated genes. This finding suggests that the repressive effects of species-specific methylation are strongest when both layers of regulation are driven in *cis*, perhaps because *cis*-regulated methylation changes tend to have larger effect sizes than *trans*-regulated changes.

Our two-step binomial sign test approach in hybrids identified multiple gene sets showing coordinated directional shifts in methylation and expression that significantly exceed neutral expectations. Many of the pathways we identified are related to known human-chimpanzee phenotypic differences: intellectual disability in hybrid DAs, growth delay, and highly arched eyebrows in hybrid iPSCs, hepatitis C in hybrid HEPs, and tooth eruption timing in parental DPSCs (Figures 5 and 6, Figure 5—figure supplement 1). Specifically, the evidence rejecting a neutral null model of *cis*-regulatory evolution consists of statistically significant directional bias in both promoter methylation and gene expression within functionally coherent gene sets, assessed using a two-step binomial sign test with permutation-based FDR correction. These findings represent the first evidence for coordinated directional divergence in DNA methylation and identify candidate genes under lineage-specific selection. By restricting analysis to genes showing negative methylation-expression associations—where methylation most likely exerts direct transcriptional repression—we identified high-confidence candidates where epigenetic divergence has likely functional consequences. Our results suggest that *cis*-regulatory changes in DNA methylation represent an important substrate for adaptive evolution, complementing protein-coding and other *cis*-regulatory changes in generating human-specific traits.

We developed several methodological innovations tailored to the unique challenges of comparative epigenomics. First, our beta-binomial framework accounts for the count-based nature of bisulfite sequencing data and explicitly models allele-specific patterns in hybrid vs. parental contexts. Second, our changepoint detection algorithm identifies coherent DMRs with minimal regulatory heterogeneity, prioritizing functionally organized regulatory units over isolated CpG sites. Third, the two-step sign test we introduce provides a method to identify lineage-specific selection that links methylation and corresponding changes in gene expression. These methods can be readily applied to other taxa where hybrid organisms can be generated.

However, our study also has important limitations. First, while hybrid cells provide powerful controls for *trans*-acting variation, in vitro differentiation protocols do not fully recapitulate the complexity and temporal dynamics of in vivo development. Therefore, cell lines are limited in their ability to capture species-specific methylation or expression profiles that are specific to a certain developmental stage or in vivo context. Second, pathway analysis of DPSCs used primary human and chimpanzee samples rather than hybrids, with *trans*-acting factors potentially inflating statistical significance in the sign test; these results should therefore be interpreted not as evidence for positive selection, but rather as an interesting pattern that could have important connections to phenotypic differences between humans and chimpanzees. Third, while we identified associations between TF binding motifs and *trans*-DMRs, definitive demonstration of causality requires targeted experimental perturbations such as base editing (for CpG-disrupting SNVs) or TF knockdown/overexpression experiments (for *trans*-acting TFs). Fourth, our study examined a limited number of cell types, and methylation divergence in most tissues remains unexplored. Fifth, our study lacks an outgroup species; therefore, we cannot definitively attribute observed differences to the human lineage specifically, and our use of the term ‘human-specific’ refers to differences between humans and chimpanzees rather than changes unique to the human lineage. Sixth, while we focused on CpG SNVs as a mechanism for *cis*-methylation divergence, other classes of variants—such as TF binding site mutations—could also influence local methylation patterns and represent an important avenue for future investigation.

Comparative gene expression analyses between humans and closely related species offer a powerful way to explore the molecular basis of species-specific traits and disease susceptibilities. However, it remains challenging to infer phenotypic differences through the lens of human clinical ontologies. To start with, HPO terms are largely derived from pathological human data and predominantly capture phenotypic consequences of LOF mutations in a clinical context. When comparing expression levels between species, one might assume that lower expression corresponds to an LOF-like phenotype. However, the relationship between expression dosage and function is typically nonlinear (Milind et al., 2024), and complete gene knockouts in a model organism or LOF mutations in patients are not equivalent to moderate downregulation. This fact may help explain cases where our sign test results suggest the opposite directionality as compared to human/chimpanzee phenotypic differences (Figure 6E). Alternatively, humans and chimpanzees may achieve a similar phenotype through distinct configurations of regulatory networks. For example, an adaptive downregulation of a particular gene may be compensated for by upregulation of other genes in the same pathway, maintaining overall pathway function. In other words, compensatory changes may complicate the relationship between gene expression and phenotype, as may be the case for the ‘poor speech’ gene set (Figure 6E; Agoglia et al., 2021).

Several important directions emerge from this work. First, extending this framework to additional cell types and developmental time points would reveal whether the predominance of *cis*-regulation we observe is universal or varies across developmental contexts. Longitudinal sampling during differentiation could identify critical windows when methylation patterns are established and reveal whether human-chimpanzee differences emerge gradually or through discrete developmental transitions. Second, integrating chromatin accessibility (ATAC-seq) and histone modification (ChIP-seq) data would provide a more complete picture of the regulatory landscape, clarifying how DNA methylation interacts with other chromatin features to establish gene expression patterns. Third, direct examination of TF binding using techniques like CUT&RUN in both species could validate the predicted relationships between TF occupancy and methylation patterns at *trans*-DMRs. Finally, extending these analyses to include other great apes as an outgroup would provide phylogenetic resolution to distinguish changes that occurred in the human vs. the chimpanzee lineage.

Collectively, our findings provide a framework for understanding how DNA methylation divergence contributes to human-specific traits. We demonstrate that while both *cis*- and *trans*-regulatory mechanisms shape interspecies methylation differences, *cis*-acting factors predominate, thus directly linking genomic sequence variation and epigenetic divergence. The coordinated nature of many independent methylation-expression changes across phenotypically relevant pathways is consistent with epigenetic modifications being important targets of natural selection in human evolution, potentially contributing to the regulatory innovations underlying human-chimpanzee phenotypic differences.

## Materials and methods

### Cell culture and differentiation

We utilized two previously characterized human-chimpanzee hybrid iPSC lines (Hy1-30 and Hy1-25) generated through in vitro cell fusion (Agoglia et al., 2021). Human (two individuals, H20961 and H21976), chimpanzee (two individuals, C3649 and C3647), and human-chimpanzee hybrid iPSCs were maintained on Matrigel in mTeSR1 or mTeSR Plus medium (STEMCELL Technologies cat #85850 or cat #100-0276) (Agoglia et al., 2021). These lines were confirmed to be free of mycoplasma contamination, and their identity was confirmed using the RNA-sequencing data generated in this study. The Columbia Stem Cell Core Facility differentiated the iPSCs into multiple cell types, including DAs, SKMs, and HEPs, using published protocols (Wang et al., 2024b; Xu et al., 2022; Burridge et al., 2014; Chal et al., 2016; Korytnikov and Nostro, 2016; Mallanna and Duncan, 2013; Maury et al., 2015; Sharma et al., 2019; Fedele et al., 2017; Studer, 2012; Kriks et al., 2011). CNCCs were differentiated as described (Gokhman et al., 2021). Primary DPSC samples were obtained from two human and two chimpanzee donors, as described (Gokhman et al., 2021).

### RNA-sequencing and ASE analysis

All samples were cryopreserved in liquid nitrogen before RNA extraction (Milani et al., 2016). Cells were gently thawed and then washed with PBS, and cell pellets were collected via centrifugation at 1000 RPM for 5 min. RNA extraction was performed using the RNeasy Mini Kit (QIAGEN, 74104), and DNase digestion was performed using the RNase-Free DNase Set (QIAGEN, 79254) following standard protocols. RNA quality was assessed using the Agilent Bioanalyzer RNA Pico assay. Only samples with an RNA integrity number (RIN) greater than or equal to 7 were used to prepare cDNA libraries. Libraries were prepared using TruSeq Stranded mRNA kits (Illumina, 20020594) and sequenced on Illumina HiSeq 4000 to generate paired-end reads. An allele-specific expression pipeline adapted from our previous work was implemented, using dual reference genome alignment to hg38 and pantro6 to eliminate mapping bias (Agoglia et al., 2021; Gokhman et al., 2021). Reads were processed with SeqPrep, mapped using STAR, deduplicated with Picard, and corrected for allelic bias using Hornet. DESeq2 was used for differential expression analysis with FDR correction, defining significant ASE as FDR<0.05 with consistent results across both reference genomes (Agoglia et al., 2021; Gokhman et al., 2021; Wang et al., 2024b; Dobin et al., 2013; St John, 2024; Broad Institute, 2025; van de Geijn et al., 2015; Love et al., 2014). For details, see ‘Generation of allele-specific count tables’ and ‘Identifying genes with ASE’ in the Methods section in Wang et al., 2024b. For hybrid WGBS samples (DAs and CNCCs), on average, 48.2% ± 0.2% of all aligned reads are unassignable using SNPs between human and chimpanzee genome, 25.9% ± 0.2% of the reads are specific to human, and 25.1% ± 0.2% of reads are specific to chimpanzee. For hybrid RRBS samples (iPSCs, SKMs, and HEPs), on average, 88.4% ± 0.3% of all aligned reads are unassignable using SNPs between human and chimpanzee genome, 5.3% ± 0.1% of the reads are specific to human, and 5.3% ± 0.1% of reads are specific to chimpanzee. The proportion of allele-resolvable reads differs markedly between the two bisulfite assays, and this difference is attributable to fragment length and to the genomic territory each assay interrogates. A read can be assigned to a parental allele only if it spans at least one informative human-chimpanzee SNV. RRBS libraries are generated by MspI digestion and are dominated by short fragments (median aligned length 49–50 bp), and their coverage is concentrated in CGI and CpG-dense promoters, which are among the most highly conserved sequences in the genome and therefore carry few interspecies SNVs. Among the 16,845,376 unique CpG positions covered by the unphased hybrid RRBS samples, 65.1% have at least one human-chimpanzee SNV within ±50 bp—the footprint reachable by a typical RRBS read—whereas 90.8% have one within ±150 bp, the footprint of a typical WGBS read pair. Consistent with this, the aligned-length distributions of allele-resolved and unassigned reads are essentially identical in all four WGBS libraries (median 150 bp for both), whereas in all six RRBS libraries allele-resolved reads are substantially longer than unassigned reads (medians 70–75 vs. 49–50 bp, respectively).

### Bisulfite sequencing library preparation, sequencing, and mapping

DNA was extracted and processed, and WGBS and RRBS were performed at Admera Health Biopharma Services using Illumina NovaSeq 6000. WGBS libraries achieved a mean coverage of 9.68× per CpG site, covering 43.6M unique CpG positions in CNCC (hybrid) and 43.4M in CNCC (parental), 41.9M in DA (hybrid, phased), and 38.8M in DA (parental). RRBS libraries achieved a mean coverage of 5.02× per CpG site, covering 5.0M, 4.8M, and 4.5M unique CpG positions in the phased hybrid HEP, iPSC, and SKM libraries, respectively, and 3.3M, 3.4M, and 3.2M in the corresponding parental libraries. After phasing the hybrid samples, the total number of CpG sites (union) of all WGBS libraries is 48,994,320 sites, and the union of all CpG sites of RRBS libraries is 7,289,251. The union of all libraries (including WGBS and RRBS) is 49,049,052. We have also included coverage information as a table in Supplementary file 1. To minimize reference mapping bias in cross-species comparisons, reference genomes were first masked at previously identified human-chimpanzee SNV positions using bedtools maskfasta, reducing the potential for allelic mapping preferences (Wang et al., 2024b; Quinlan and Hall, 2010). Reference genome preparation was performed using Bismark’s bismark_genome_preparation with Bowtie2 as the aligner, which converts unmethylated cytosines in silico to enable bisulfite-aware alignment (Krueger and Andrews, 2011; Langmead and Salzberg, 2012). All samples were aligned to both masked human (hg38) and chimpanzee (pantro6) reference genomes to assess mapping quality and species-specific biases. Reads were quality-trimmed and adapter sequences removed using Cutadapt and Trim Galore with default parameters for RRBS libraries, including the removal of artificially filled-in cytosines from the 3' end of reads (Martin, 2011; Krueger, 2021). Trimmed reads were aligned using Bismark, and methylation levels were calculated as the ratio of unconverted cytosines to total coverage at each CpG site (Krueger and Andrews, 2011). For hybrid samples, SNPsplit was used to assign reads to either the human or chimpanzee allele (Krueger and Andrews, 2016). Strand information was collapsed for symmetric CpG sites, and genomic annotations were lifted between genomic assemblies hg38 and pantro6 using UCSC LiftOver with reciprocal mapping to ensure orthologous regions (Karolchik et al., 2004; Hinrichs et al., 2006).

### PCA and hierarchical clustering

Fractional methylation of individual CpG sites was estimated using bismark_methylation_extractor from Bismark (Krueger and Andrews, 2011). CpG sites shared across samples were identified using bedtools intersect (Quinlan and Hall, 2010). PCA was performed using PCASamples from methylKit across all samples and samples within each cell type (Akalin et al., 2012). Euclidean distance matrices were computed across all samples for hierarchical clustering analysis, with visualization performed using the pheatmap package (Kolde, 2025). Clustering patterns were assessed to validate cell-type identity and characterize species-specific methylation signatures.

### Beta-binomial regulatory classification framework

**Figure 7.**
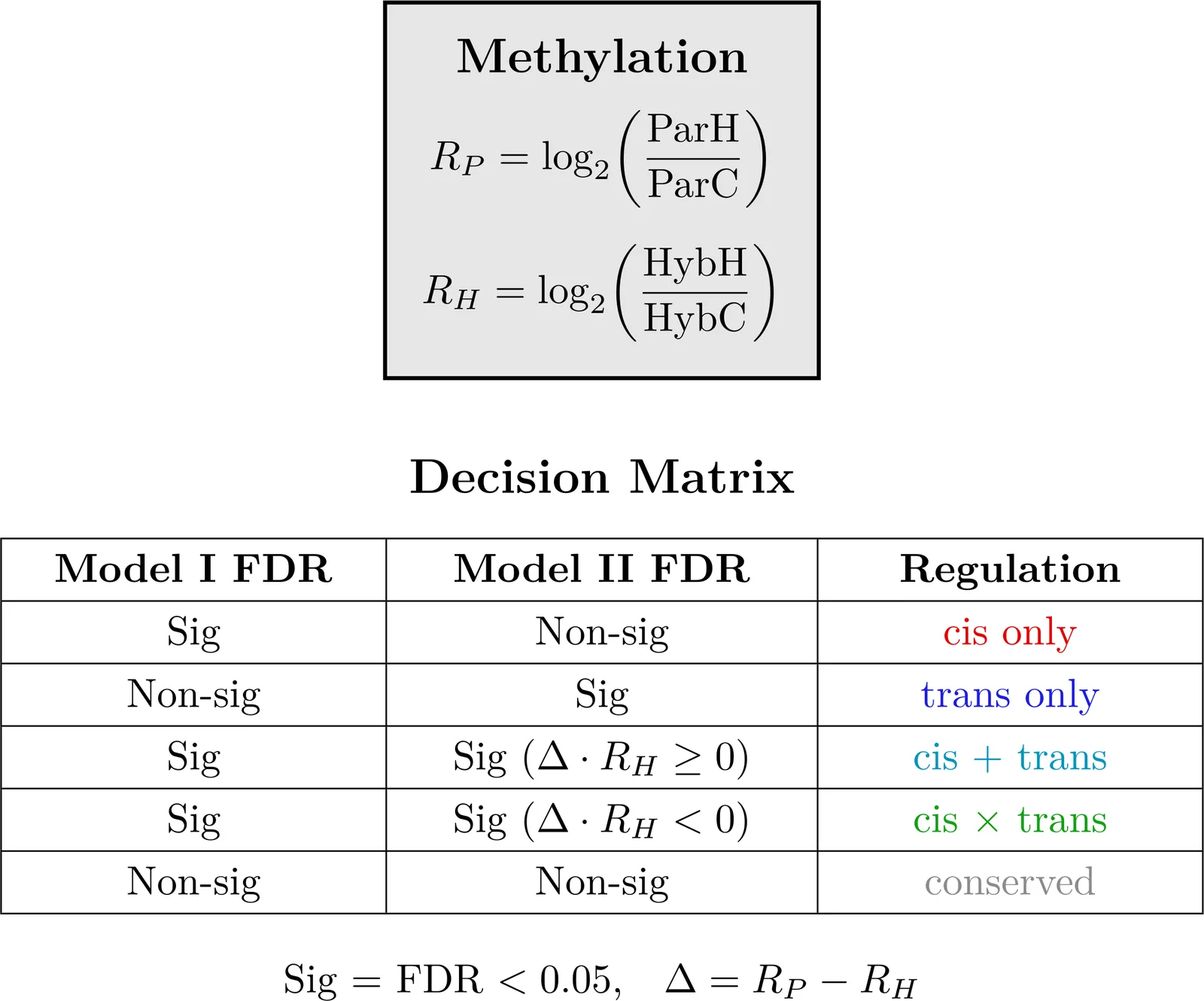
Decision rules for classifying regulatory divergence in methylation. R_P_ and R_H_ are log_2_ ratios of human to chimpanzee methylation in parental (ParH/ParC) and hybrid (HybH/HybC) samples. Model I tests for an allelic difference in hybrids (cis) and Model II tests System × Allele interaction (trans). Loci significant in both Model I and Model II are classified as cis + trans when Δ and R_H_ have the same sign (Δ·R_H_ ≥ 0) and as cis × trans when they have opposite signs. Sig, FDR < 0.05.

Methylation data from human (H) and chimpanzee (C) parental and hybrid samples were analyzed using a beta-binomial generalized linear modeling framework to account for overdispersion inherent to bisulfite sequencing count data. The data included measurements for parental H-genome (ParH), parental C-genome (ParC), hybrid H-allele (HybH), and hybrid C-allele (HybC) for each genomic locus (Figure 7). For each genomic locus (site or annotated region), methylated counts were modeled using two complementary beta-binomial generalized linear models to separately evaluate *cis*- and *trans*-regulatory effects:

**Model I:** *Cis* effect test with methylation data on hybrid alleles

$$\displaystyle {\rm logit} (\pi) = \beta_0 + \beta_1 (\rm Allele) $$

**Model II:** *Trans* effect test using System × Allele interaction term with methylation data in both hybrid and parental alleles

$$\displaystyle {\rm logit(\pi) = \beta_0 + \beta_1(\rm System) + \beta_2(\rm Allele) + \beta_3(\rm System \times Allele)}$$

where π is the probability of methylation, System distinguishes parental from hybrid samples, Allele distinguishes between human and chimpanzee methylation, and the interaction term captures any signals involving the change of allelic differences across systems. Statistical significance was assessed through likelihood ratio tests comparing nested models, testing for system effects, allele effects (*cis*-regulation), and system-by-allele interactions (*trans*-regulation). Genomic loci were classified into five categories: conserved, *trans*-only, *cis*-only, *cis* × *trans* interaction, and *cis + trans* effects:

- **Conserved**: No significant allelic differences in either system (neither parental nor hybrid allelic tests significant)
- ***Trans* only**: Significant allelic difference in parents but not hybrids, indicating *trans*-acting regulation
- ***Cis* only**: Significant allelic differences in both systems with no significant interaction, indicating consistent *cis*-acting effects
- ***Cis* × *trans***: Significant allelic differences in both systems with significant interaction, where effects occur in opposite directions
- ***Cis* + *trans***: Significant allelic differences in both systems with significant interaction, where effects are in the same direction

Regional analyses combined CpG-level methylation across defined genomic intervals (e.g. promoters). Regional methylation was quantified as pooled fractional methylation, calculated as the total number of methylated cytosines divided by the total coverage across all CpG sites within each region. Nominal p-values<0.05 from both models were used to provide a schematic overview of site-level CpG methylation in scatterplots and bar plots, preserving the full dynamic range of the data and providing input features for downstream de novo DMR identification. For reporting significant candidate loci under each regulation category, as well as formal inference of region-level regulation, Benjamini-Hochberg FDR was calculated to account for multiple testing and to evaluate the robustness of inferred relative contribution of regulatory mechanisms across significance thresholds (Benjamini and Hochberg, 1995). The same classification and visualization steps were employed using FDR-corrected p-values on loci and regions to confirm that the relative contributions of regulatory classifications were not driven by significance cutoffs alone (Figure 2—figure supplements 1 and 2). To assess the robustness of *cis*-over-*trans* contributions to methylation with respect to the significance cutoffs, the proportion of significant loci and regions assigned to each regulatory category was estimated across a range of FDR cutoffs (FDR<0.01, 0.05, 0.1, 0.2, 0.25), confirming that the observed *cis* dominance over other divergent regulation categories (including *trans*) was robust to the choice of cutoff (Figure 2—figure supplements 1 and 2).

### Changepoint detection for identifying DMRs with homogeneous *cis*- or *trans*-acting methylation divergence

To identify clusters of CpG sites in the same regulation category, we employed the Pruned Exact Linear Time (PELT) algorithm implemented in the changepoint R package to detect shifts in mean methylation scores within each chromosome segment while accounting for a manually specified penalty against overfitting (Killick et al., 2012; Haynes et al., 2017; Yokoyama et al., 2015). We adapted this method to identify regions in the genome where neighboring CpG sites are consistently classified by the previously mentioned beta-binomial model to fall under the same regulatory classification. We first segmented the entire genome into large regions where a break is assigned whenever neighboring CpG sites are more than 5000 bp apart. Then, we transform all CpG sites in a segment into binary sequences, where the focal regulation category is 1 and alternative categories are 0. To identify *trans*-DMRs, we set focal pure *trans*-regulation to 1, and all other regulation categories to 0. To identify *cis*-DMRs, we set focal pure *cis*-regulation to 1, and all other regulation categories to 0. To identify *trans-*DMRs specifically for HOMER enrichment inputs, we used a relaxed gradient scoring approach. We set focal pure *trans*-regulation to 1, conserved to 0.75, additive (*cis + trans*) and interactive (*cis* × *trans*) regulation to 0.5, and pure *cis*-regulation category to 0. To determine the optimal penalty parameter, we applied the CROPS (Changepoints for a Range Of Penalties) method, testing penalty values ranging from 0.01 to 2.0. For each penalty range giving the same number of segments, we recorded the number of detected changepoints and corresponding model fit statistics. The optimal penalty for each segment was determined using elbow point detection via the R package kneedle, which identifies the point of maximum distance from the line connecting endpoints in the penalty-vs.-changepoints curve (Satopaa et al., 2011). We then applied a homogeneity filter requiring segments to have a minimum target proportion of 0.6, where the target proportion is the fraction of CpG sites within the segment that (1) belong to the target regulatory class under the beta-binomial *cis*/*trans* classification and (2) share a consistent species bias in methylation (all human-biased or all chimpanzee-biased), to ensure biological relevance of identified regions. For segments where fewer than three distinct penalty values yielded different numbers of changepoints, we selected the penalty that maximized the number of segments passing the homogeneity threshold. Segments without detected changepoints were evaluated directly against the homogeneity criterion to assess their potential as single-segment DMRs.

To refine segment boundaries and remove spurious edge effects before merging overlapping DMRs, we implemented a post-processing trimming step that removes segment edges containing sites with regulatory patterns inconsistent with the dominant signal within each segment. This ensures that identified DMRs represent coherent regions of coordinated methylation changes rather than artifacts of the segmentation process. *cis*-DMRs, *trans*-DMRs, *cis* × *trans*, and *cis + trans-*DMRs were identified independently by applying the changepoint detection method separately to each regulatory class.

### Identification of cell-type-specific DMRs, species-specific DMRs, and cell-type-specific species-specific DMRs

Examples of cell-type-specific DMRs (*HOXA9*), species-specific DMRs (*CTSF*), and cell-type-specific, species-specific DMRs (*LGALS8*) were identified in hybrid samples using the DSS-single (Dispersion Shrinkage for Sequencing data) (Wu et al., 2013; Feng et al., 2014; Wu et al., 2015; Park and Wu, 2016). For cell-type-specific DMR identification, we used the formula ~ species + cell type + species:cell type to test for cell-type-specific methylation differences, while species-specific DMRs analysis employed ~species to identify consistent species differences across cell types. Downstream identification of cell-type-specific species-specific DMRs identifies the regions where the interaction term species:cell type is significant. The DSS analysis incorporated smoothing across neighboring CpG sites with a smoothing span of 500 bp and required a minimum of five CpG sites per region. Statistical significance was determined using Wald tests with FDR<0.05, and effect size filtering retained regions with methylation difference ≥20%. Integration with gene expression data was performed through Wilcoxon rank-sum tests to identify cell-type-specific gene expression patterns associated with cell-type-specific DMRs, while permutations (n=1000) were used to assess the significance of coordinated methylation-expression changes in species-specific patterns, which accounts for any background correlation between neighboring genomic features.

### Expression-methylation correlation analysis

Human transcript coordinates and gene body annotations were obtained from the hg38.knownGene.gtf.gz file via UCSC Table Browser (Karolchik et al., 2004), with transcript start positions designated as TSS. CpG methylation data aggregated within ENCODE-defined promoter regions were analyzed using a beta-binomial classification framework to determine regulatory categories, while corresponding gene expression data underwent parallel hypothesis testing using a negative-binomial model (Hallgrímsdóttir et al., 2024; Abascal et al., 2020). Genes with the same classification for both methylation and expression—both *cis* or both *trans*—were then analyzed separately. For genes within each regulatory category, the 2 kb surrounding each TSS was split into 10 bins of 200 bp where individual CpG sites were pooled and averaged as fractional methylation values and correlated with expression TPM values. Custom scripts used for binning and correlation are adapted from beta_profile_region.py from CpGtools (Wei et al., 2021).

### Sign test selection analysis

To identify coordinated methylation-expression changes potentially under selection, we used a binomial sign test framework that accounts for background probabilities of concordant changes (Agoglia et al., 2021; Gokhman et al., 2021; Wang et al., 2024b; Starr and Fraser, 2025; Orr, 1998; Simon et al., 2024). Promoter regions were obtained from the ENCODE Project Consortium (Abascal et al., 2020). Canonical transcripts were downloaded from the UCSC Table Browser hg38 assembly. For genes with multiple annotated promoters, we selected the promoter that is the closest to the 5' UTR of the gene, to ensure each gene is assigned one corresponding promoter (Karolchik et al., 2004). Promoter regions were mapped to canonical transcripts, and functional enrichment using pathways from the KEGG and HPO was assessed using directional sign tests that leverage the independence of *cis*-regulatory regions to detect non-random patterns indicative of lineage-specific selection rather than neutral drift (Fraser, 2013; Agoglia et al., 2021; Gokhman et al., 2021; Wang et al., 2024b; Fraser, 2011; Sharon et al., 2018; Artieri and Fraser, 2014; Nourmohammad et al., 2017; Papakostas et al., 2014). For the methylation-only sign test, gene sets with more than 200 genes or fewer than 20 genes with corresponding promoter methylation data were excluded from the analysis. Lineage-specific selection on a gene set was inferred if there was a statistically significant directionality bias in chimpanzee vs. human promoter ASM. For the sign test on genes with putatively repressive methylation—i.e., genes where increased methylation within 1 kb of their TSS corresponds to decreased expression—we first classified genes as either consistent with repressive methylation (either Ch>Hu methylation paired with Hu>Ch expression, or Hu>Ch methylation paired with Ch>Hu expression) or not consistent. Gene sets with more than 200 genes or fewer than 10 genes with corresponding methylation and expression data were excluded from the analysis. We then reran the sign test using a new background probability defined as the overall fraction of genes with repressive methylation in each cell type. FDRs of the resulting binomial p-values were estimated by randomly permuting the signs of all genes and performing the binomial sign test 10^4^ times. To calculate FDR, we compared each pathway’s empirical binomial p-value against the distribution of permuted p-values. The expected number of false positives was estimated as the average number of pathways reaching that significance level per permutation. FDR was then defined as this expected count of false positives divided by the observed count of pathways with empirical p-values at least as extreme.

### *cis-*DMR sequence variant analysis and *trans-*DMR motif enrichment analysis

High-confidence human-chimpanzee SNVs were identified using a dual-reference approach with stringent filtering criteria as described previously (Wang et al., 2024b). ASM within different genomic ranges (25 bp, 50 bp, 100 bp) of CpG-altering variants was calculated. Proportions of regions with Hu>Ch and Hu<Ch methylation were calculated for SNVs in three categories: SNVs that disrupt CpG dinucleotides in humans, SNVs that disrupt CpG sites in chimpanzees, and SNVs that lead to no interspecies changes in CpG content between the species. The significance of the difference in relative proportions of Hu>Ch and Hu<Ch of SNVs in the three categories was assessed using a two-proportion test. For proximity tests, the Mann-Whitney U test (Mann and Whitney, 1947) was used to compare the distribution of distances from all *cis*- or all *trans*-DMRs to the closest CpG SNV. HOMER’s findMotifsGenome.pl was used to test for motif enrichment in the *trans*-DMR set using the human reference genome hg38 as background (Heinz et al., 2010).

## Data Availability

Raw and processed data generated by this study are publicly available through the Gene Expression Omnibus under accession GSE315270 (BS-seq) and GSE315269 (RNA-seq). The raw and processed bulk RNA-seq data of hybrid and parental iPSCs by Agoglia et al. is available at https://www.ncbi.nlm.nih.gov/geo/query/acc.cgi?acc=GSE144825 and the raw and processed bulk RNA-seq data of hybrid and parental CNCC by Gokhman et al. is available at https://www.ncbi.nlm.nih.gov/geo/query/acc.cgi?acc=GSE146481. The raw and processed bulk RNA-seq data of hybrid hepatocytes and hybrid skeletal myocytes from Wang et al. are available at https://www.ncbi.nlm.nih.gov/geo/query/acc.cgi?acc=GSE232949. scRNA-seq data of parental hepatocytes and parental skeletal myocytes from Barr et al. are available here: https://www.ncbi.nlm.nih.gov/geo/query/acc.cgi?acc=GSE201516. The log fold-changes and associated statistics were used directly from the supplemental material Data S1 of Barr et al. The human and chimpanzee reference genomes used are available at https://hgdownload.soe.ucsc.edu/goldenPath/hg38/bigZips/ and https://hgdownload.soe.ucsc.edu/goldenPath/panTro6/bigZips/ respectively. The human-chimp pairwise alignment used to identify SNVs and indels is available at https://hgdownload.soe.ucsc.edu/goldenPath/hg38/vsPanTro6/. All scripts for performing analyses and making figures in this manuscript are available at https://github.com/zhenzhenma120/The-causes-and-consequences-of-human-specific-DNA-methylation, copy archived at Zhenzhen, 2026. All supplementary files are available on Zenodo at https://doi.org/10.5281/zenodo.24116290. The following datasets were generated: MaZ StarrA GokhmanD FraserH 2026The causes and consequences of human-specific DNA methylation [WGBS; RRBS]NCBI Gene Expression OmnibusGSE315270 MaZ StarrA GokhmanD FraserH 2026The causes and consequences of human-specific DNA methylation [RNA-Seq]NCBI Gene Expression OmnibusGSE315269 MaZ StarrA GokhmanD FraserH 2026Supplementary Files for publication "Cis-regulation and natural selection in the evolution of human DNA methylation"Zenodo10.5281/zenodo.24116290PMC1375918643018124 The following previously published datasets were used: AgogliaR FraserH PascaS 2020Primate cell fusion disentangles gene regulatory divergence in neural developmentNCBI Gene Expression OmnibusGSE14482510.1038/s41586-021-03343-3PMC871963333731928 GokhmanD FraserH 2020Human-chimpanzee hybrid cells reveal gene regulatory evolution underlying skeletal divergenceNCBI Gene Expression OmnibusGSE146481 WangB StarrA FraserH 2023Cell type-specific cis-regulatory divergence in gene expression and chromatin accessibility revealed by human-chimpanzee hybrid cellsNCBI Gene Expression OmnibusGSE23294910.7554/eLife.89594PMC1094260838358392 BarrK RhodesK GiladY 2023The relationship between regulatory changes in cis and trans and the evolution of gene expression in humans and chimpanzeesNCBI Gene Expression OmnibusGSE20151610.1186/s13059-023-03019-3PMC1049617137697401

## Additional files

MDAR checklist

## References

[bib1] Abascal F, Acosta R, Addleman NJ, Adrian J, Afzal V, Ai R, Aken B, Akiyama JA, Jammal OA, Amrhein H, Anderson SM, Andrews GR, Antoshechkin I, Ardlie KG, Armstrong J, Astley M, Banerjee B, Barkal AA, Barnes IHA, Barozzi I, Barrell D, Barson G, Bates D, Baymuradov UK, Bazile C, Beer MA, Beik S, Bender MA, Bennett R, Bouvrette LPB, Bernstein BE, Berry A, Bhaskar A, Bignell A, Blue SM, Bodine DM, Boix C, Boley N, Borrman T, Borsari B, Boyle AP, Brandsmeier LA, Breschi A, Bresnick EH, Brooks JA, Buckley M, Burge CB, Byron R, Cahill E, Cai L, Cao L, Carty M, Castanon RG, Castillo A, Chaib H, Chan ET, Chee DR, Chee S, Chen H, Chen H, Chen JY, Chen S, Cherry JM, Chhetri SB, Choudhary JS, Chrast J, Chung D, Clarke D, Cody NAL, Coppola CJ, Coursen J, D’Ippolito AM, Dalton S, Danyko C, Davidson C, Davila-Velderrain J, Davis CA, Dekker J, Deran A, DeSalvo G, Despacio-Reyes G, Dewey CN, Dickel DE, Diegel M, Diekhans M, Dileep V, Ding B, Djebali S, Dobin A, Dominguez D, Donaldson S, Drenkow J, Dreszer TR, Drier Y, Duff MO, Dunn D, Eastman C, Ecker JR, Edwards MD, El-Ali N, Elhajjajy SI, Elkins K, Emili A, Epstein CB, Evans RC, Ezkurdia I, Fan K, Farnham PJ, Farrell NP, Feingold EA, Ferreira AM, Fisher-Aylor K, Fitzgerald S, Flicek P, Foo CS, Fortier K, Frankish A, Freese P, Fu S, Fu XD, Fu Y, Fukuda-Yuzawa Y, Fulciniti M, Funnell APW, Gabdank I, Galeev T, Gao M, Giron CG, Garvin TH, Gelboin-Burkhart CA, Georgolopoulos G, Gerstein MB, Giardine BM, Gifford DK, Gilbert DM, Gilchrist DA, Gillespie S, Gingeras TR, Gong P, Gonzalez A, Gonzalez JM, Good P, Goren A, Gorkin DU, Graveley BR, Gray M, Greenblatt JF, Griffiths E, Groudine MT, Grubert F, Gu M, Guigó R, Guo H, Guo Y, Guo Y, Gursoy G, Gutierrez-Arcelus M, Halow J, Hardison RC, Hardy M, Hariharan M, Harmanci A, Harrington A, Harrow JL, Hashimoto TB, Hasz RD, Hatan M, Haugen E, Hayes JE, He P, He Y, Heidari N, Hendrickson D, Heuston EF, Hilton JA, Hitz BC, Hochman A, Holgren C, Hou L, Hou S, Hsiao YHE, Hsu S, Huang H, Hubbard TJ, Huey J, Hughes TR, Hunt T, Ibarrientos S, Issner R, Iwata M, Izuogu O, Jaakkola T, Jameel N, Jansen C, Jiang L, Jiang P, Johnson A, Johnson R, Jungreis I, Kadaba M, Kasowski M, Kasparian M, Kato M, Kaul R, Kawli T, Kay M, Keen JC, Keles S, Keller CA, Kelley D, Kellis M, Kheradpour P, Kim DS, Kirilusha A, Klein RJ, Knoechel B, Kuan S, Kulik MJ, Kumar S, Kundaje A, Kutyavin T, Lagarde J, Lajoie BR, Lambert NJ, Lazar J, Lee AY, Lee D, Lee E, Lee JW, Lee K, Leslie CS, Levy S, Li B, Li H, Li N, Li S, Li X, Li YI, Li Y, Li Y, Li Y, Lian J, Libbrecht MW, Lin S, Lin Y, Liu D, Liu J, Liu P, Liu T, Liu XS, Liu Y, Liu Y, Long M, Lou S, Loveland J, Lu A, Lu Y, Lécuyer E, Ma L, Mackiewicz M, Mannion BJ, Mannstadt M, Manthravadi D, Marinov GK, Martin FJ, Mattei E, McCue K, McEown M, McVicker G, Meadows SK, Meissner A, Mendenhall EM, Messer CL, Meuleman W, Meyer C, Miller S, Milton MG, Mishra T, Moore DE, Moore HM, Moore JE, Moore SH, Moran J, Mortazavi A, Mudge JM, Munshi N, Murad R, Myers RM, Nandakumar V, Nandi P, Narasimha AM, Narayanan AK, Naughton H, Navarro FCP, Navas P, Nazarovs J, Nelson J, Neph S, Neri FJ, Nery JR, Nesmith AR, Newberry JS, Newberry KM, Ngo V, Nguyen R, Nguyen TB, Nguyen T, Nishida A, Noble WS, Novak CS, Novoa EM, Nuñez B, O’Donnell CW, Olson S, Onate KC, Otterman E, Ozadam H, Pagan M, Palden T, Pan X, Park Y, Partridge EC, Paten B, Pauli-Behn F, Pazin MJ, Pei B, Pennacchio LA, Perez AR, Perry EH, Pervouchine DD, Phalke NN, Pham Q, Phanstiel DH, Plajzer-Frick I, Pratt GA, Pratt HE, Preissl S, Pritchard JK, Pritykin Y, Purcaro MJ, Qin Q, Quinones-Valdez G, Rabano I, Radovani E, Raj A, Rajagopal N, Ram O, Ramirez L, Ramirez RN, Rausch D, Raychaudhuri S, Raymond J, Razavi R, Reddy TE, Reimonn TM, Ren B, Reymond A, Reynolds A, Rhie SK, Rinn J, Rivera M, Rivera-Mulia JC, Roberts BS, Rodriguez JM, Rozowsky J, Ryan R, Rynes E, Salins DN, Sandstrom R, Sasaki T, Sathe S, Savic D, Scavelli A, Scheiman J, Schlaffner C, Schloss JA, Schmitges FW, See LH, Sethi A, Setty M, Shafer A, Shan S, Sharon E, Shen Q, Shen Y, Sherwood RI, Shi M, Shin S, Shoresh N, Siebenthall K, Sisu C, Slifer T, Sloan CA, Smith A, Snetkova V, Snyder MP, Spacek DV, Srinivasan S, Srivas R, Stamatoyannopoulos G, Stamatoyannopoulos JA, Stanton R, Steffan D, Stehling-Sun S, Strattan JS, Su A, Sundararaman B, Suner MM, Syed T, Szynkarek M, Tanaka FY, Tenen D, Teng M, Thomas JA, Toffey D, Tress ML, Trout DE, Trynka G, Tsuji J, Upchurch SA, Ursu O, Uszczynska-Ratajczak B, Uziel MC, Valencia A, Biber BV, van der Velde AG, Van Nostrand EL, Vaydylevich Y, Vazquez J, Victorsen A, Vielmetter J, Vierstra J, Visel A, Vlasova A, Vockley CM, Volpi S, Vong S, Wang H, Wang M, Wang Q, Wang R, Wang T, Wang W, Wang X, Wang Y, Watson NK, Wei X, Wei Z, Weisser H, Weissman SM, Welch R, Welikson RE, Weng Z, Westra HJ, Whitaker JW, White C, White KP, Wildberg A, Williams BA, Wine D, Witt HN, Wold B, Wolf M, Wright J, Xiao R, Xiao X, Xu J, Xu J, Yan KK, Yan Y, Yang H, Yang X, Yang YW, Yardımcı GG, Yee BA, Yeo GW, Young T, Yu T, Yue F, Zaleski C, Zang C, Zeng H, Zeng W, Zerbino DR, Zhai J, Zhan L, Zhan Y, Zhang B, Zhang J, Zhang J, Zhang K, Zhang L, Zhang P, Zhang Q, Zhang XO, Zhang Y, Zhang Z, Zhao Y, Zheng Y, Zhong G, Zhou XQ, Zhu Y, Zimmerman J, Moore JE, Purcaro MJ, Pratt HE, Epstein CB, Shoresh N, Adrian J, Kawli T, Davis CA, Dobin A, Kaul R, Halow J, Van Nostrand EL, Freese P, Gorkin DU, Shen Y, He Y, Mackiewicz M, Pauli-Behn F, Williams BA, Mortazavi A, Keller CA, Zhang XO, Elhajjajy SI, Huey J, Dickel DE, Snetkova V, Wei X, Wang X, Rivera-Mulia JC, Rozowsky J, Zhang J, Chhetri SB, Zhang J, Victorsen A, White KP, Visel A, Yeo GW, Burge CB, Lécuyer E, Gilbert DM, Dekker J, Rinn J, Mendenhall EM, Ecker JR, Kellis M, Klein RJ, Noble WS, Kundaje A, Guigó R, Farnham PJ, Cherry JM, Myers RM, Ren B, Graveley BR, Gerstein MB, Pennacchio LA, Snyder MP, Bernstein BE, Wold B, Hardison RC, Gingeras TR, Stamatoyannopoulos JA, Weng Z (2020). Expanded encyclopaedias of DNA elements in the human and mouse genomes. Nature.

[bib2] Agoglia RM, Sun D, Birey F, Yoon S-J, Miura Y, Sabatini K, Pașca SP, Fraser HB (2021). Primate cell fusion disentangles gene regulatory divergence in neurodevelopment. Nature.

[bib3] Akalin A, Kormaksson M, Li S, Garrett-Bakelman FE, Figueroa ME, Melnick A, Mason CE (2012). methylKit: a comprehensive R package for the analysis of genome-wide DNA methylation profiles. Genome Biology.

[bib4] Artieri CG, Fraser HB (2014). Evolution at two levels of gene expression in yeast. Genome Research.

[bib5] Bai X, Cao R, Wu D, Zhang H, Yang F, Wang L (2023). Dental pulp stem cells for bone tissue engineering: a literature review. Stem Cells International.

[bib6] Banerjee N, Polushina T, Bettella F, Steen VM, Andreassen OA, Le Hellard S (2019). Analysis of differentially methylated regions in great apes and extinct hominids provides support for the evolutionary hypothesis of schizophrenia. Schizophrenia Research.

[bib7] Barr KA, Rhodes KL, Gilad Y (2023). The relationship between regulatory changes in cis and trans and the evolution of gene expression in humans and chimpanzees. Genome Biology.

[bib8] Benjamini Y, Hochberg Y (1995). Controlling the false discovery rate: a practical and powerful approach to multiple testing. Journal of the Royal Statistical Society Series B.

[bib9] Blake LE, Roux J, Hernando-Herraez I, Banovich NE, Perez RG, Hsiao CJ, Eres I, Cuevas C, Marques-Bonet T, Gilad Y (2020). A comparison of gene expression and DNA methylation patterns across tissues and species. Genome Research.

[bib10] Bockenhauer D, Jaureguiberry G (2016). HNF1B-associated clinical phenotypes: the kidney and beyond. Pediatric Nephrology.

[bib11] Boman J, Qvarnström A, Mugal CF (2024). Regulatory and evolutionary impact of DNA methylation in two songbird species and their naturally occurring F_1_ hybrids. BMC Biology.

[bib12] Braccioli L, Nijboer CH, Coffer PJ (2018). Forkhead box protein P1, a key player in neuronal development?. Neural Regeneration Research.

[bib13] (2025). Github.

[bib14] Bronner ME, Simões-Costa M (2016). The neural crest migrating into the twenty-first century. Current Topics in Developmental Biology.

[bib15] Burridge PW, Matsa E, Shukla P, Lin ZC, Churko JM, Ebert AD, Lan F, Diecke S, Huber B, Mordwinkin NM, Plews JR, Abilez OJ, Cui B, Gold JD, Wu JC (2014). Chemically defined generation of human cardiomyocytes. Nature Methods.

[bib16] Cassimeris L, Spittle C (2001). Regulation of microtubule-associated proteins. International Review of Cytology.

[bib17] Chal J, Al Tanoury Z, Hestin M, Gobert B, Aivio S, Hick A, Cherrier T, Nesmith AP, Parker KK, Pourquié O (2016). Generation of human muscle fibers and satellite-like cells from human pluripotent stem cells in vitro. Nature Protocols.

[bib18] Cordero DR, Brugmann S, Chu Y, Bajpai R, Jame M, Helms JA (2011). Cranial neural crest cells on the move: their roles in craniofacial development. American Journal of Medical Genetics. Part A.

[bib19] Davison RJ, Gurven MD (2021). Human uniqueness? Life history diversity among small-scale societies and chimpanzees. PLOS ONE.

[bib20] Dean MC (2006). Tooth microstructure tracks the pace of human life-history evolution. Proceedings. Biological Sciences.

[bib21] Dixon AD, Hoyte DAN, Rönning O (2017). Fundamentals of Craniofacial Growth.

[bib22] Dobin A, Davis CA, Schlesinger F, Drenkow J, Zaleski C, Jha S, Batut P, Chaisson M, Gingeras TR (2013). STAR: ultrafast universal RNA-seq aligner. Bioinformatics.

[bib23] Ee L-S, Medina-Cano D, Goetzler E, Uyehara C, Schwarz C, Salataj E, Madhuranath S, Evans T, Hadjantonakis A-K, Apostolou E, Polyzos A, Vierbuchen T, Stadtfeld M (2025). Enhancer remodeling by OTX2 directs specification and patterning of mammalian definitive endoderm. Developmental Cell.

[bib24] Enard W, Fassbender A, Model F, Adorján P, Pääbo S, Olek A (2004). Differences in DNA methylation patterns between humans and chimpanzees. Current Biology.

[bib25] Fedele S, Collo G, Behr K, Bischofberger J, Müller S, Kunath T, Christensen K, Gündner AL, Graf M, Jagasia R, Taylor V (2017). Expansion of human midbrain floor plate progenitors from induced pluripotent stem cells increases dopaminergic neuron differentiation potential. Scientific Reports.

[bib26] Feng H, Conneely KN, Wu H (2014). A Bayesian hierarchical model to detect differentially methylated loci from single nucleotide resolution sequencing data. Nucleic Acids Research.

[bib27] Fraser HB (2011). Genome‐wide approaches to the study of adaptive gene expression evolution. BioEssays.

[bib28] Fraser HB (2013). Gene expression drives local adaptation in humans. Genome Research.

[bib29] Fukuda K, Ichiyanagi K, Yamada Y, Go Y, Udono T, Wada S, Maeda T, Soejima H, Saitou N, Ito T, Sasaki H (2013). Regional DNA methylation differences between humans and chimpanzees are associated with genetic changes, transcriptional divergence and disease genes. Journal of Human Genetics.

[bib30] Gargano MA, Matentzoglu N, Coleman B, Addo-Lartey EB, Anagnostopoulos AV, Anderton J, Avillach P, Bagley AM, Bakštein E, Balhoff JP, Baynam G, Bello SM, Berk M, Bertram H, Bishop S, Blau H, Bodenstein DF, Botas P, Boztug K, Čady J, Callahan TJ, Cameron R, Carbon SJ, Castellanos F, Caufield JH, Chan LE, Chute CG, Cruz-Rojo J, Dahan-Oliel N, Davids JR, de Dieuleveult M, de Souza V, de Vries BBA, de Vries E, DePaulo JR, Derfalvi B, Dhombres F, Diaz-Byrd C, Dingemans AJM, Donadille B, Duyzend M, Elfeky R, Essaid S, Fabrizzi C, Fico G, Firth HV, Freudenberg-Hua Y, Fullerton JM, Gabriel DL, Gilmour K, Giordano J, Goes FS, Moses RG, Green I, Griese M, Groza T, Gu W, Guthrie J, Gyori B, Hamosh A, Hanauer M, Hanušová K, He YO, Hegde H, Helbig I, Holasová K, Hoyt CT, Huang S, Hurwitz E, Jacobsen JOB, Jiang X, Joseph L, Keramatian K, King B, Knoflach K, Koolen DA, Kraus ML, Kroll C, Kusters M, Ladewig MS, Lagorce D, Lai M-C, Lapunzina P, Laraway B, Lewis-Smith D, Li X, Lucano C, Majd M, Marazita ML, Martinez-Glez V, McHenry TH, McInnis MG, McMurry JA, Mihulová M, Millett CE, Mitchell PB, Moslerová V, Narutomi K, Nematollahi S, Nevado J, Nierenberg AA, Čajbiková NN, Nurnberger JI, Ogishima S, Olson D, Ortiz A, Pachajoa H, Perez de Nanclares G, Peters A, Putman T, Rapp CK, Rath A, Reese J, Rekerle L, Roberts AM, Roy S, Sanders SJ, Schuetz C, Schulte EC, Schulze TG, Schwarz M, Scott K, Seelow D, Seitz B, Shen Y, Similuk MN, Simon ES, Singh B, Smedley D, Smith CL, Smolinsky JT, Sperry S, Stafford E, Stefancsik R, Steinhaus R, Strawbridge R, Sundaramurthi JC, Talapova P, Tenorio Castano JA, Tesner P, Thomas RH, Thurm A, Turnovec M, van Gijn ME, Vasilevsky NA, Vlčková M, Walden A, Wang K, Wapner R, Ware JS, Wiafe AA, Wiafe SA, Wiggins LD, Williams AE, Wu C, Wyrwoll MJ, Xiong H, Yalin N, Yamamoto Y, Yatham LN, Yocum AK, Young AH, Yüksel Z, Zandi PP, Zankl A, Zarante I, Zvolský M, Toro S, Carmody LC, Harris NL, Munoz-Torres MC, Danis D, Mungall CJ, Köhler S, Haendel MA, Robinson PN (2024). The human phenotype ontology in 2024: phenotypes around the world. Nucleic Acids Research.

[bib31] Gerdes J, Lemke H, Baisch H, Wacker HH, Schwab U, Stein H (1984). Cell cycle analysis of a cell proliferation-associated human nuclear antigen defined by the monoclonal antibody Ki-67. Journal of Immunology.

[bib32] Gertz J, Varley KE, Reddy TE, Bowling KM, Pauli F, Parker SL, Kucera KS, Willard HF, Myers RM (2011). Analysis of DNA methylation in a three-generation family reveals widespread genetic influence on epigenetic regulation. PLOS Genetics.

[bib33] Gokhman D, Nissim-Rafinia M, Agranat-Tamir L, Housman G, García-Pérez R, Lizano E, Cheronet O, Mallick S, Nieves-Colón MA, Li H, Alpaslan-Roodenberg S, Novak M, Gu H, Osinski JM, Ferrando-Bernal M, Gelabert P, Lipende I, Mjungu D, Kondova I, Bontrop R, Kullmer O, Weber G, Shahar T, Dvir-Ginzberg M, Faerman M, Quillen EE, Meissner A, Lahav Y, Kandel L, Liebergall M, Prada ME, Vidal JM, Gronostajski RM, Stone AC, Yakir B, Lalueza-Fox C, Pinhasi R, Reich D, Marques-Bonet T, Meshorer E, Carmel L (2020). Differential DNA methylation of vocal and facial anatomy genes in modern humans. Nature Communications.

[bib34] Gokhman D, Agoglia RM, Kinnebrew M, Gordon W, Sun D, Bajpai VK, Naqvi S, Chen C, Chan A, Chen C, Petrov DA, Ahituv N, Zhang H, Mishina Y, Wysocka J, Rohatgi R, Fraser HB (2021). Human-chimpanzee fused cells reveal cis-regulatory divergence underlying skeletal evolution. Nature Genetics.

[bib35] Gosalia N, Yang R, Kerschner JL, Harris A (2015). FOXA2 regulates a network of genes involved in critical functions of human intestinal epithelial cells. Physiological Genomics.

[bib36] Graffmann N, Scherer B, Adjaye J (2022). In vitro differentiation of pluripotent stem cells into hepatocyte like cells - basic principles and current progress. Stem Cell Research.

[bib37] Grozdanov PN, Yovchev MI, Dabeva MD (2006). The oncofetal protein glypican-3 is a novel marker of hepatic progenitor/oval cells. Laboratory Investigation; A Journal of Technical Methods and Pathology.

[bib38] Guevara EE, Lawler RR, Staes N, White CM, Sherwood CC, Ely JJ, Hopkins WD, Bradley BJ (2020). Age-associated epigenetic change in chimpanzees and humans. Philosophical Transactions of the Royal Society B.

[bib39] Hallgrímsdóttir IB, Carilli M, Pachter L (2024). Estimating cis and trans contributions to differences in gene regulation. bioRxiv.

[bib40] Hatori A, Fujii Y, Kawase-Koga Y, Ogasawara T, Chikira J, Minami S, Yamakawa D, Chikazu D (2023). VCAM-1 and GFPT-2: predictive markers of osteoblast differentiation in human dental pulp stem cells. Bone.

[bib41] Haynes K, Eckley IA, Fearnhead P (2017). Computationally efficient changepoint detection for a range of penalties. Journal of Computational and Graphical Statistics.

[bib42] Heinz S, Benner C, Spann N, Bertolino E, Lin YC, Laslo P, Cheng JX, Murre C, Singh H, Glass CK (2010). Simple combinations of lineage-determining transcription factors prime cis-regulatory elements required for macrophage and B cell identities. Molecular Cell.

[bib43] Hellman A, Chess A (2010). Extensive sequence-influenced DNA methylation polymorphism in the human genome. Epigenetics & Chromatin.

[bib44] Hernando-Herraez I, Prado-Martinez J, Garg P, Fernandez-Callejo M, Heyn H, Hvilsom C, Navarro A, Esteller M, Sharp AJ, Marques-Bonet T (2013). Dynamics of DNA methylation in recent human and great ape evolution. PLOS Genetics.

[bib45] Hernando-Herraez I, Garcia-Perez R, Sharp AJ, Marques-Bonet T (2015a). DNA methylation: insights into human evolution. PLOS Genetics.

[bib46] Hernando-Herraez I, Heyn H, Fernandez-Callejo M, Vidal E, Fernandez-Bellon H, Prado-Martinez J, Sharp AJ, Esteller M, Marques-Bonet T (2015b). The interplay between DNA methylation and sequence divergence in recent human evolution. Nucleic Acids Research.

[bib47] Hinrichs AS, Karolchik D, Baertsch R, Barber GP, Bejerano G, Clawson H, Diekhans M, Furey TS, Harte RA, Hsu F, Hillman-Jackson J, Kuhn RM, Pedersen JS, Pohl A, Raney BJ, Rosenbloom KR, Siepel A, Smith KE, Sugnet CW, Sultan-Qurraie A, Thomas DJ, Trumbower H, Weber RJ, Weirauch M, Zweig AS, Haussler D, Kent WJ (2006). The UCSC genome browser database: update 2006. Nucleic Acids Research.

[bib48] Housman G, Quillen EE, Stone AC (2021). An evolutionary perspective of DNA methylation patterns in skeletal tissues using a baboon model of osteoarthritis. Journal of Orthopaedic Research.

[bib49] Hu CK, York RA, Metz HC, Bedford NL, Fraser HB, Hoekstra HE (2022). cis-Regulatory changes in locomotor genes are associated with the evolution of burrowing behavior. Cell Reports.

[bib50] Iwafuchi-Doi M, Donahue G, Kakumanu A, Watts JA, Mahony S, Pugh BF, Lee D, Kaestner KH, Zaret KS (2016). The pioneer transcription factor foxa maintains an accessible nucleosome configuration at enhancers for tissue-specific gene activation. Molecular Cell.

[bib51] Jaenisch R, Bird A (2003). Epigenetic regulation of gene expression: how the genome integrates intrinsic and environmental signals. Nature Genetics.

[bib52] Jones JH (2011). Primates and the evolution of long, slow life histories. Current Biology.

[bib53] Jones PA (2012). Functions of DNA methylation: islands, start sites, gene bodies and beyond. Nature Reviews. Genetics.

[bib54] Kanehisa M, Goto S (2000). KEGG: kyoto encyclopedia of genes and genomes. Nucleic Acids Research.

[bib55] Karolchik D, Hinrichs AS, Furey TS, Roskin KM, Sugnet CW, Haussler D, Kent WJ (2004). The UCSC table browser data retrieval tool. Nucleic Acids Research.

[bib56] Kelley JL, Gilad Y (2020). Effective study design for comparative functional genomics. Nature Reviews. Genetics.

[bib57] Killick R, Fearnhead P, Eckley IA (2012). Optimal detection of changepoints with a linear computational cost. Journal of the American Statistical Association.

[bib58] King MC, Wilson AC (1975). Evolution at two levels in humans and chimpanzees. Science.

[bib59] Kolde R (2025). Github.

[bib60] Korytnikov R, Nostro MC (2016). Generation of polyhormonal and multipotent pancreatic progenitor lineages from human pluripotent stem cells. Methods.

[bib61] Kriks S, Shim J-W, Piao J, Ganat YM, Wakeman DR, Xie Z, Carrillo-Reid L, Auyeung G, Antonacci C, Buch A, Yang L, Beal MF, Surmeier DJ, Kordower JH, Tabar V, Studer L (2011). Dopamine neurons derived from human ES cells efficiently engraft in animal models of Parkinson’s disease. Nature.

[bib62] Krueger F, Andrews SR (2011). Bismark: a flexible aligner and methylation caller for bisulfite-seq applications. Bioinformatics.

[bib63] Krueger F, Andrews SR (2016). SNPsplit: Allele-specific splitting of alignments between genomes with known SNP genotypes. F1000Research.

[bib64] Krueger F (2021). Github.

[bib65] Lalmansingh AS, Karmakar S, Jin Y, Nagaich AK (2012). Multiple modes of chromatin remodeling by Forkhead box proteins. Biochimica et Biophysica Acta- Gene Regulatory Mechanisms.

[bib66] Langmead B, Salzberg SL (2012). Fast gapped-read alignment with Bowtie 2. Nature Methods.

[bib67] Lee K, Cho H, Rickert RW, Li QV, Pulecio J, Leslie CS, Huangfu D (2019). FOXA2 is required for enhancer priming during pancreatic differentiation. Cell Reports.

[bib68] Li Y, Banerjee S, Wang Y, Goldstein SA, Dong B, Gaughan C, Silverman RH, Weiss SR (2016). Activation of RNase L is dependent on OAS3 expression during infection with diverse human viruses. PNAS.

[bib69] Li J, Wu X, Ke J, Lee M, Lan Q, Li J, Yu J, Huang Y, Sun DQ, Xie R (2022). TET1 dioxygenase is required for FOXA2-associated chromatin remodeling in pancreatic beta-cell differentiation. Nature Communications.

[bib70] Love MI, Huber W, Anders S (2014). Moderated estimation of fold change and dispersion for RNA-seq data with DESeq2. Genome Biology.

[bib71] Ma S, Skarica M, Li Q, Xu C, Risgaard RD, Tebbenkamp ATN, Mato-Blanco X, Kovner R, Krsnik Ž, de Martin X, Luria V, Martí-Pérez X, Liang D, Karger A, Schmidt DK, Gomez-Sanchez Z, Qi C, Gobeske KT, Pochareddy S, Debnath A, Hottman CJ, Spurrier J, Teo L, Boghdadi AG, Homman-Ludiye J, Ely JJ, Daadi EW, Mi D, Daadi M, Marín O, Hof PR, Rasin MR, Bourne J, Sherwood CC, Santpere G, Girgenti MJ, Strittmatter SM, Sousa AMM, Sestan N (2022). Molecular and cellular evolution of the primate dorsolateral prefrontal cortex. Science.

[bib72] Mack KL, Nachman MW (2017). Gene regulation and speciation. Trends in Genetics.

[bib73] Mack KL, Talbott HE, Griffin MF, Parker JBL, Guardino NJ, Spielman AF, Davitt MF, Mascharak S, Downer M, Morgan A, Valencia C, Akras D, Berger MJ, Wan DC, Fraser HB, Longaker MT (2023). Allele-specific expression reveals genetic drivers of tissue regeneration in mice. Cell Stem Cell.

[bib74] Mallanna SK, Duncan SA (2013). Differentiation of hepatocytes from pluripotent stem cells. Current Protocols in Stem Cell Biology.

[bib75] Mann HB, Whitney DR (1947). On a test of whether one of two random variables is stochastically larger than the other. The Annals of Mathematical Statistics.

[bib76] Martin M (2011). Cutadapt removes adapter sequences from high-throughput sequencing reads. EMBnet.Journal.

[bib77] Maury Y, Côme J, Piskorowski RA, Salah-Mohellibi N, Chevaleyre V, Peschanski M, Martinat C, Nedelec S (2015). Combinatorial analysis of developmental cues efficiently converts human pluripotent stem cells into multiple neuronal subtypes. Nature Biotechnology.

[bib78] Mendizabal I, Shi L, Keller TE, Konopka G, Preuss TM, Hsieh T-F, Hu E, Zhang Z, Su B, Yi SV (2016). Comparative methylome analyses identify epigenetic regulatory loci of human brain evolution. Molecular Biology and Evolution.

[bib79] Metzakopian E, Lin W, Salmon-Divon M, Dvinge H, Andersson E, Ericson J, Perlmann T, Whitsett JA, Bertone P, Ang S-L (2012). Genome-wide characterization of Foxa2 targets reveals upregulation of floor plate genes and repression of ventrolateral genes in midbrain dopaminergic progenitors. Development.

[bib80] Milani P, Escalante-Chong R, Shelley BC, Patel-Murray NL, Xin X, Adam M, Mandefro B, Sareen D, Svendsen CN, Fraenkel E (2016). Cell freezing protocol suitable for ATAC-Seq on motor neurons derived from human induced pluripotent stem cells. Scientific Reports.

[bib81] Milind N, Smith CJ, Zhu H, Gjorgjieva T, Spence JP, Pritchard JK (2024). Buffering and Non-Monotonic Behavior of Gene Dosage Response Curves for Human Complex Traits. bioRxiv.

[bib82] Murrell A, Rakyan VK, Beck S (2005). From genome to epigenome. Human Molecular Genetics.

[bib83] Noor A, Windpassinger C, Patel M, Stachowiak B, Mikhailov A, Azam M, Irfan M, Siddiqui ZK, Naeem F, Paterson AD, Lutfullah M, Vincent JB, Ayub M (2008). CC2D2A, encoding a coiled-coil and C2 domain protein, causes autosomal-recessive mental retardation with retinitis pigmentosa. American Journal of Human Genetics.

[bib84] Nourmohammad A, Rambeau J, Held T, Kovacova V, Berg J, Lässig M (2017). Adaptive evolution of gene expression in drosophila. Cell Reports.

[bib85] Orr HA (1998). Testing natural selection vs. genetic drift in phenotypic evolution using quantitative trait locus data. Genetics.

[bib86] Pai AA, Bell JT, Marioni JC, Pritchard JK, Gilad Y (2011). A genome-wide study of DNA methylation patterns and gene expression levels in multiple human and chimpanzee tissues. PLOS Genetics.

[bib87] Papakostas S, Vøllestad LA, Bruneaux M, Aykanat T, Vanoverbeke J, Ning M, Primmer CR, Leder EH (2014). Gene pleiotropy constrains gene expression changes in fish adapted to different thermal conditions. Nature Communications.

[bib88] Park Y, Wu H (2016). Differential methylation analysis for BS-seq data under general experimental design. Bioinformatics.

[bib89] Poirier K, Saillour Y, Bahi-Buisson N, Jaglin XH, Fallet-Bianco C, Nabbout R, Castelnau-Ptakhine L, Roubertie A, Attie-Bitach T, Desguerre I, Genevieve D, Barnerias C, Keren B, Lebrun N, Boddaert N, Encha-Razavi F, Chelly J (2010). Mutations in the neuronal ß-tubulin subunit TUBB3 result in malformation of cortical development and neuronal migration defects. Human Molecular Genetics.

[bib90] Prud’homme B, Gompel N, Carroll SB (2007). Emerging principles of regulatory evolution. PNAS.

[bib91] Quinlan AR, Hall IM (2010). BEDTools: a flexible suite of utilities for comparing genomic features. Bioinformatics.

[bib92] Reilly SK, Noonan JP (2016). Evolution of gene regulation in humans. Annual Review of Genomics and Human Genetics.

[bib93] Reizel Y, Morgan A, Gao L, Schug J, Mukherjee S, García MF, Donahue G, Baur JA, Zaret KS, Kaestner KH (2021). FoxA-dependent demethylation of DNA initiates epigenetic memory of cellular identity. Developmental Cell.

[bib94] Romano R, Bucci C (2020). Role of EGFR in the nervous system. Cells.

[bib95] Romero IG, Ruvinsky I, Gilad Y (2012). Comparative studies of gene expression and the evolution of gene regulation. Nature Reviews. Genetics.

[bib96] Rooryck C, Diaz-Font A, Osborn DPS, Chabchoub E, Hernandez-Hernandez V, Shamseldin H, Kenny J, Waters A, Jenkins D, Kaissi AA, Leal GF, Dallapiccola B, Carnevale F, Bitner-Glindzicz M, Lees M, Hennekam R, Stanier P, Burns AJ, Peeters H, Alkuraya FS, Beales PL (2011). Mutations in lectin complement pathway genes COLEC11 and MASP1 cause 3MC syndrome. Nature Genetics.

[bib97] Rubenstein AB, Smith GR, Raue U, Begue G, Minchev K, Ruf-Zamojski F, Nair VD, Wang X, Zhou L, Zaslavsky E, Trappe TA, Trappe S, Sealfon SC (2020). Single-cell transcriptional profiles in human skeletal muscle. Scientific Reports.

[bib98] Satopaa V, Albrecht J, Irwin D, Raghavan B (2011). Finding a ‘kneedle’ in a haystack: detecting knee points in system behavior.

[bib99] Saxonov S, Berg P, Brutlag DL (2006). A genome-wide analysis of CpG dinucleotides in the human genome distinguishes two distinct classes of promoters. PNAS.

[bib100] Sérandour AA, Avner S, Percevault F, Demay F, Bizot M, Lucchetti-Miganeh C, Barloy-Hubler F, Brown M, Lupien M, Métivier R, Salbert G, Eeckhoute J (2011). Epigenetic switch involved in activation of pioneer factor FOXA1-dependent enhancers. Genome Research.

[bib101] Sharma R, Khristov V, Rising A, Jha BS, Dejene R, Hotaling N, Li Y, Stoddard J, Stankewicz C, Wan Q, Zhang C, Campos MM, Miyagishima KJ, McGaughey D, Villasmil R, Mattapallil M, Stanzel B, Qian H, Wong W, Chase L, Charles S, McGill T, Miller S, Maminishkis A, Amaral J, Bharti K (2019). Clinical-grade stem cell-derived retinal pigment epithelium patch rescues retinal degeneration in rodents and pigs. Science Translational Medicine.

[bib102] Sharon E, Chen S-AA, Khosla NM, Smith JD, Pritchard JK, Fraser HB (2018). Functional genetic variants revealed by massively parallel precise genome editing. Cell.

[bib103] Silva J, Nichols J, Theunissen TW, Guo G, van Oosten AL, Barrandon O, Wray J, Yamanaka S, Chambers I, Smith A (2009). Nanog is the gateway to the pluripotent ground state. Cell.

[bib104] Simon NM, Kim Y, Gribnau J, Bautista DM, Dutton JR, Brem RB (2024). Stem cell transcriptional profiles from mouse subspecies reveal cis-regulatory evolution at translation genes. Heredity.

[bib105] Smith TM, Machanda Z, Bernard AB, Donovan RM, Papakyrikos AM, Muller MN, Wrangham R (2013). First molar eruption, weaning, and life history in living wild chimpanzees. PNAS.

[bib106] Soldatov R, Kaucka M, Kastriti ME, Petersen J, Chontorotzea T, Englmaier L, Akkuratova N, Yang Y, Häring M, Dyachuk V, Bock C, Farlik M, Piacentino ML, Boismoreau F, Hilscher MM, Yokota C, Qian X, Nilsson M, Bronner ME, Croci L, Hsiao W-Y, Guertin DA, Brunet J-F, Consalez GG, Ernfors P, Fried K, Kharchenko PV, Adameyko I (2019). Spatiotemporal structure of cell fate decisions in murine neural crest. Science.

[bib107] Song JHT, Grant RL, Behrens VC, Kučka M, Roberts Kingman GA, Soltys V, Chan YF, Kingsley DM (2021). Genetic studies of human–chimpanzee divergence using stem cell fusions. PNAS.

[bib108] Song JHT, Carter AC, Bushinsky EM, Beck SG, Petrocelli JE, Koreman GT, Babu J, Kingsley DM, Greenberg ME, Walsh CA (2025). Human-chimpanzee tetraploid system defines mechanisms of species-specific neural gene regulation. bioRxiv.

[bib109] Starr AL, Gokhman D, Fraser HB (2023). Accounting for cis-regulatory constraint prioritizes genes likely to affect species-specific traits. Genome Biology.

[bib110] Starr AL, Fraser HB (2025). A general principle of neuronal evolution reveals a human-accelerated neuron type potentially underlying the high prevalence of autism in humans. Molecular Biology and Evolution.

[bib111] Stein R, Razin A, Cedar H (1982). In vitro methylation of the hamster adenine phosphoribosyltransferase gene inhibits its expression in mouse L cells. PNAS.

[bib112] St John J (2024). Github.

[bib113] Stolz JR, Foote KM, Veenstra-Knol HE, Pfundt R, ten Broeke SW, de Leeuw N, Roht L, Pajusalu S, Part R, Rebane I, Õunap K, Stark Z, Kirk EP, Lawson JA, Lunke S, Christodoulou J, Louie RJ, Rogers RC, Davis JM, Innes AM, Wei XC, Keren B, Mignot C, Lebel RR, Sperber SM, Sakonju A, Dosa N, Barge-Schaapveld D, Peeters-Scholte C, Ruivenkamp CAL, van Bon BW, Kennedy J, Low KJ, Ellard S, Pang L, Junewick JJ, Mark PR, Carvill GL, Swanson GT (2021). Clustered mutations in the GRIK2 kainate receptor subunit gene underlie diverse neurodevelopmental disorders. The American Journal of Human Genetics.

[bib114] Studer L (2012). Derivation of dopaminergic neurons from pluripotent stem cells. Progress in Brain Research.

[bib115] Svandova E, Vesela B, Kratochvilova A, Holomkova K, Oralova V, Dadakova K, Burger T, Sharpe P, Lesot H, Matalova E (2023). Markers of dental pulp stem cells in in vivo developmental context. Annals of Anatomy - Anatomischer Anzeiger.

[bib116] Teh M-T, Gemenetzidis E, Patel D, Tariq R, Nadir A, Bahta AW, Waseem A, Hutchison IL (2012). FOXM1 induces a global methylation signature that mimics the cancer epigenome in head and neck squamous cell carcinoma. PLOS ONE.

[bib117] Tischfield MA, Baris HN, Wu C, Rudolph G, Van Maldergem L, He W, Chan W-M, Andrews C, Demer JL, Robertson RL, Mackey DA, Ruddle JB, Bird TD, Gottlob I, Pieh C, Traboulsi EI, Pomeroy SL, Hunter DG, Soul JS, Newlin A, Sabol LJ, Doherty EJ, de Uzcátegui CE, de Uzcátegui N, Collins MLZ, Sener EC, Wabbels B, Hellebrand H, Meitinger T, de Berardinis T, Magli A, Schiavi C, Pastore-Trossello M, Koc F, Wong AM, Levin AV, Geraghty MT, Descartes M, Flaherty M, Jamieson RV, Møller HU, Meuthen I, Callen DF, Kerwin J, Lindsay S, Meindl A, Gupta ML, Pellman D, Engle EC (2010). Human TUBB3 mutations perturb microtubule dynamics, kinesin interactions, and axon guidance. Cell.

[bib118] Valbuena S, Lerma J (2021). Kainate receptors, homeostatic gatekeepers of synaptic plasticity. Neuroscience.

[bib119] van de Geijn B, McVicker G, Gilad Y, Pritchard JK (2015). WASP: allele-specific software for robust molecular quantitative trait locus discovery. Nature Methods.

[bib120] Vilgalys TP, Rogers J, Jolly CJ, Mukherjee S, Tung J (2019). Evolution of DNA methylation in papio baboons. Molecular Biology and Evolution.

[bib121] Walker CM (1997). Comparative features of hepatitis C virus infection in humans and chimpanzees. Springer Seminars in Immunopathology.

[bib122] Wang J, Liu X, Lan Y, Que T, Li J, Yue B, Fan Z (2024a). DNA methylation and transcriptome analysis reveal epigenomic differences among three macaque species. Evolutionary Applications.

[bib123] Wang B, Starr AL, Fraser HB (2024b). Cell-type-specific *cis*-regulatory divergence in gene expression and chromatin accessibility revealed by human-chimpanzee hybrid cells. eLife.

[bib124] Wei T, Nie J, Larson NB, Ye Z, Eckel-Passow JE, Robertson KD, Kocher JPA, Wang L (2021). CpGtools: a python package for DNA methylation analysis. Bioinformatics.

[bib125] Wittkopp PJ, Kalay G (2012). Cis-regulatory elements: molecular mechanisms and evolutionary processes underlying divergence. Nature Reviews Genetics.

[bib126] Wu H, Wang C, Wu Z (2013). A new shrinkage estimator for dispersion improves differential expression detection in RNA-seq data. Biostatistics.

[bib127] Wu H, Xu T, Feng H, Chen L, Li B, Yao B, Qin Z, Jin P, Conneely KN (2015). Detection of differentially methylated regions from whole-genome bisulfite sequencing data without replicates. Nucleic Acids Research.

[bib128] Xu P, He H, Gao Q, Zhou Y, Wu Z, Zhang X, Sun L, Hu G, Guan Q, You Z, Zhang X, Zheng W, Xiong M, Chen Y (2022). Human midbrain dopaminergic neuronal differentiation markers predict cell therapy outcomes in a Parkinson’s disease model. The Journal of Clinical Investigation.

[bib129] Yee LJ, Tang YM, Kleiner DE, Wang D, Im K, Wahed A, Tong X, Rhodes S, Su X, Whelan MR, Fontana RJ, Ghany MG, Borg B, Liang JT, Yang H (2007). Myxovirus-1 and protein kinase haplotypes and fibrosis in chronic hepatitis C virus. Hepatology.

[bib130] Yokoyama T, Miura F, Araki H, Okamura K, Ito T (2015). Changepoint detection in base-resolution methylome data reveals a robust signature of methylated domain landscape. BMC Genomics.

[bib131] Zeng J, Konopka G, Hunt BG, Preuss TM, Geschwind D, Yi SV (2012). Divergent whole-genome methylation maps of human and chimpanzee brains reveal epigenetic basis of human regulatory evolution. American Journal of Human Genetics.

[bib132] Zhenzhen M (2026). Software Heritage.

[bib133] Zhi D, Aslibekyan S, Irvin MR, Claas SA, Borecki IB, Ordovas JM, Absher DM, Arnett DK (2013). SNPs located at CpG sites modulate genome-epigenome interaction. Epigenetics.

