# Supplementary material for: Cis-regulation and natural selection in the evolution of human DNA methylation
Source: eLife. 2026 Nov 12;15:RP110575. doi: 10.7554/eLife.110575 (PMC13759186; doi:10.7554/eLife.110575)
Supplement: Peer review history

# Editor report: eLife Assessment

**DOI:** 10.7554/eLife.110575.4.sa0

**Authors:** Jenny Tung

**Affiliations:** Max Planck Institute for Evolutionary Anthropology Germany

**Keywords:** Compelling, Important

This study presents an **important** examination of the role of cis-acting versus trans-acting genetic variation in DNA methylation divergence between humans and chimpanzees and its relationship to gene expression. By differentiating fused interspecies tetraploid cell lines into multiple cell types, the study provides **compelling** evidence that cis-acting changes predominate in DNA methylation divergence. The evidence linking directional methylation divergence to lineage-specific selection and, particularly, to adaptive human phenotypes is less clear. This work will be of interest to biologists and evolutionary anthropologists studying the evolution and genetics of gene regulation, particularly in primates.

# Referee report: Reviewer #1 (Public review)

**DOI:** 10.7554/eLife.110575.4.sa1

[Editor's note: this version has been assessed by the Reviewing Editor without further input from the original reviewers. The authors have addressed the comments raised in the previous round of review.]

Ma et al. use human-chimpanzee tetraploid cells, across different cell types, to identify the genetic causes and then transcriptomic consequences of divergence in DNA methylation. They conclude that the evolution of DNA methylation is driven primarily by cis-regulatory changes, and that the evolution of CpG sites contributes to cis-regulation while transcription factor expression underlies some trans changes. They then argue that divergence in DNA methylation is associated with changes in gene expression and may contribute to human phenotypes.

The tetraploid model is able to provide compelling evidence that most regulatory evolution occurs due to cis-regulatory changes, and that sites that have diverged in DNA methylation level cluster together into DMRs which share similar divergence patterns and genetic architecture. This stands in intriguing contrast to trans-mechanisms which explain most variation within modern human populations. The authors proceed to show that trans-effects can be explained, in part, by nearby TF binding motifs and many cis-regulatory changes are explained by CpG-disrupting variants. While these mechanisms clearly contribution to divergence in DNA methylation, the degree to which they explain cis- and trans-regulatory divergence would be valuable.

Next, the authors seek to show that differences in DNA methylation are functionally relevant. Consistent with previous results, they show that differences in DNA methylation are (weakly) associated with changes in gene expression. They hypothesize that genes with concordant regulatory should exhibit great methylation-expression coupling than other genes and show that cis-expression/cis-methylation pairs are more strongly correlated than trans/trans pairs. I think that looking at cis/trans or trans/cis changes would also be useful. Another limitation is that this analysis is limited to promoter regions. It is not clear how many divergent DMRs that includes and how many of those genes have differences in expression. The key question is whether differences in DNA methylation are functionally important, and the answer provided by these analyses is "sometimes".

Finally, the authors make a case for lineage-specific selection on DNA methylation that is connected to human traits. Using a sign-test, they identify sets of genes which have likely experienced selection in humans and may underlie human phenotypes.

In conclusion, I think this study provides a valuable resource for differences in DNA methylation between humans and chimpanzees across tissues and provides important insight into the relative abundance of cis and trans regulatory divergence. Additional research is necessary to investigate the underlying regulatory mechanisms and more care needs to be taken in exploring the functional consequences.

# Referee report: Reviewer #3 (Public review)

**DOI:** 10.7554/eLife.110575.4.sa2

Summary:

Ma et al. use human-chimpanzee tetraploid cells to examine species differences in DNA methylation. They identify differentially methylated regions under cis or trans regulation. Cis-DMRs are enriched near SNVs that disrupt or create CpGs, providing a plausible mechanism for cis changes in methylation. They also seek to identify transcription factors that might affect methylation in trans, as well as gene sets with evidence for consistent changes in methylation and expression between humans and chimpanzees, suggesting that they may underlie lineage-specific traits.

Strengths:

The authors have generated a new dataset across multiple different cell types examining differences in DNA methylation between humans and chimpanzees using human diploid cells, chimpanzee diploid cells, and human-chimpanzee tetraploid cells. Using this dataset, they identify that cis-DMRs are enriched near SNVs that disrupt or create CpGs compared to trans-DMRs and identify transcription factors as candidate trans-acting factors. Both identified SNVs and transcription factors are good candidates for future experimentation. The authors also find that cis-DMRs are more highly correlated with cis-expressed genes than trans-DMRs with trans-expressed genes, providing evidence that methylation and expression are linked genome-wide. Further, they apply a clever sign test approach to identify gene pathways with evidence for lineage-specific selection.

Comments on revised version.

They authors have addressed my concerns from their initial manuscript. In particular, the authors have noted the limitations of their study as appropriate, including the use of permissive FDR cut-offs.

# Author comment: Author response

**DOI:** 10.7554/eLife.110575.4.sa3

**Authors:** Zhenzhen Ma, Alexander L Starr, David Gokhman, Hunter B Fraser

**Affiliations:** Stanford University Palo Alto United States; Stanford University Stanford United States; Weizmann Institute of Science Rehovot Israel; Stanford University Stanford United States

**Public Reviews:**

**Reviewer #1 (Public review):**

Ma et al. use human-chimpanzee tetraploid cells, across different cell types, to identify the genetic causes and then transcriptomic consequences of divergence in DNA methylation. They conclude that the evolution of DNA methylation is driven primarily by cis-regulatory changes, and that the evolution of CpG sites contributes to cis-regulation while transcription factor expression underlies some trans changes. They then argue that divergence in DNA methylation is associated with changes in gene expression and may contribute to human phenotypes.

The tetraploid model is able to provide compelling evidence that most regulatory evolution occurs due to cis-regulatory changes, and that sites that have diverged in DNA methylation level cluster together into DMRs which share similar divergence patterns and genetic architecture. This stands in intriguing contrast to trans-mechanisms which explain most variation within modern human populations. The authors proceed to show that trans-effects can be explained, in part, by nearby TF binding motifs and many cis-regulatory changes are explained by CpG-disrupting variants. While these mechanisms clearly contribution to divergence in DNA methylation, the degree to which they explain cis- and trans-regulatory divergence would be valuable.

Next, the authors seek to show that differences in DNA methylation are functionally relevant. Consistent with previous results, they show that differences in DNA methylation are (weakly) associated with changes in gene expression. They hypothesize that genes with concordant regulatory should exhibit great methylation-expression coupling than other genes and show that cis-expression/cis-methylation pairs are more strongly correlated than trans/trans pairs. I think that looking at cis/trans or trans/cis changes would also be useful. Another limitation is that this analysis is limited to promoter regions. It is not clear how many divergent DMRs that includes and how many of those genes have differences in expression. The key question is whether differences in DNA methylation are functionally important, and the answer provided by these analyses is "sometimes".

Finally, the authors make a case for lineage-specific selection on DNA methylation that is connected to human traits. Using a sign-test, they identify sets of genes which have likely experienced selection in humans and may underlie human phenotypes.

In conclusion, I think this study provides a valuable resource for differences in DNA methylation between humans and chimpanzees across tissues and provides important insight into the relative abundance of cis and trans regulatory divergence. Additional research is necessary to investigate the underlying regulatory mechanisms and more care needs to be taken in exploring the functional consequences.

**Reviewer #3 (Public review):**

Summary:

Ma et al. use human-chimpanzee tetraploid cells to examine species differences in DNA methylation. They identify differentially methylated regions under cis or trans regulation. Cis-DMRs are enriched near SNVs that disrupt or create CpGs, providing a plausible mechanism for cis changes in methylation. They also seek to identify transcription factors that might affect methylation in trans, as well as gene sets with evidence for consistent changes in methylation and expression between humans and chimpanzees, suggesting that they may underlie lineage-specific traits.

Strengths:

The authors have generated a new dataset across multiple different cell types examining differences in DNA methylation between humans and chimpanzees using human diploid cells, chimpanzee diploid cells, and human-chimpanzee tetraploid cells. Using this dataset, they identify that cis-DMRs are enriched near SNVs that disrupt or create CpGs compared to trans-DMRs and identify transcription factors as candidate trans-acting factors. Both identified SNVs and transcription factors are good candidates for future experimentation. The authors also find that cis-DMRs are more highly correlated with cis-expressed genes than trans-DMRs with trans-expressed genes, providing evidence that methylation and expression are linked genome-wide. Further, they apply a clever sign test approach to identify gene pathways with evidence for lineage-specific selection.

Comments on revised version.

They authors have addressed my concerns from their initial manuscript. In particular, the authors have noted the limitations of their study as appropriate, including the use of permissive FDR cut-offs.

We thank the Reviewing Editor, Senior Editor, and reviewers for their careful reading of our manuscript and their constructive feedback.' We have made two changes in response to the 2nd round of reviews.

We have replaced the paragraph “To identify the molecular mechanisms driving methylation divergence, we analyzed transcription factors (TFs) associated with trans-DMRs and sequence variants associated with cis-DMRs” with “To explore potential molecular mechanisms driving methylation divergence, we focused on two of many possible contributors: transcription factors (TFs) associated with trans-DMRs and sequence variants associated with cis-DMRs.”

We now report the absolute numbers of sites underlying each proportion: at FDR < 0.05, 663,368 cis-regulated and 140,017 trans-regulated sites out of 932,573 divergent sites, among 24,935,901 CpG sites tested across cell types. Only CpG sites covered in all parental and hybrid samples of a cell type can be tested, so this is fewer than the 49,049,052 unique CpG positions covered by any library, and we have clarified this distinction in the Results. Per-cell-type counts are provided in Supplementary file 1.

Second, we performed a variance partitioning analysis analogous to heritability partitioning. We decomposed total interspecies divergence (parental human minus chimpanzee) into a cis component (the allelic difference within hybrids) and a trans component (the remainder), and estimated the proportion of variance in divergence explained by each, together with their covariance. To limit sampling noise, we pooled read counts within 1-kb genomic bins. We used noise-corrected estimators based on covariances between independent replicates. For cell types with a single parental line per species, we report bounds, whose validity we confirmed in CNCC, where two parental replicates allow a direct replicate-based estimate.

In CNCC, cis effects explained 93% (95% CI 92–95%) of the variance in divergence and trans effects 71% (70–73%), with a strongly negative covariance term (−0.64). This indicates that cis and trans changes often act in opposite directions. In the other cell types, cis effects explained 58–98% and trans effects 3–48% of the variance, depending on cell type and bound, with small covariance terms. Cis exceeded trans in every cell type under both bounds, and the results were robust to a stricter coverage threshold and consistent at single-CpG resolution.

These analyses confirm that cis-regulation is the dominant contributor to human–chimpanzee methylation divergence. They also show that trans effects contribute more to divergence variance than the proportions of classified sites alone would suggest. We have added these results to the Results, described the method in a new Methods subsection, "Variance partitioning of methylation divergence”, and provided full results in Supplementary file 1.
